# Deubiquitinase USP13 regulates glycolytic reprogramming and progression in osteosarcoma by stabilizing METTL3/m^6^A/ATG5 axis

**DOI:** 10.7150/ijbs.82081

**Published:** 2023-04-23

**Authors:** Ce Wang, Yichen Meng, Jianquan Zhao, Jun Ma, Yuechao Zhao, Rui Gao, Wei Liu, Xuhui Zhou

**Affiliations:** 1 Department of Orthopedics, Changzheng Hospital, Second Affiliated Hospital of Naval Medical University, Shanghai, China; 2 Department of Orthopedics, Shanghai General Hospital, Shanghai Jiaotong University School of Medicine, Shanghai, China

**Keywords:** glycolytic reprogramming, USP13, METTL3, ATG5, N^6^-methyladenosine

## Abstract

Reprogramming metabolism is a hallmark of cancer cells for rapid progression. However, the detailed functional role of deubiquitinating enzymes (DUBs) in tumor glycolytic reprogramming is still unknown and requires further investigation. USP13 was found to upregulate in osteosarcoma (OS) specimens and promote OS progression through regulating aerobic glycolysis. Interestingly, the m^6^A writer protein, METTL3, has been identified as a novel target of USP13. USP13 interacts with, deubiquitinates, and therefore stabilizes METTL3 at K488 by removing K48-linked ubiquitin chains. Since METTL3 is a well-known m^6^A writer and USP13 stabilizes METTL3, we further found that USP13 increased global m^6^A abundance in OS cells. The results of RNA sequencing and methylated RNA immunoprecipitation sequencing indicated METTL3 could bind to m^6^A-modified ATG5 mRNA, which is crucial for autophagosome formation, and inhibit ATG5 mRNA decay on an IGF2BP3 dependent manner, thereby promoting autophagy and the autophagy-associated malignancy of OS. Using a small-molecule inhibitor named Spautin-1 to pharmacologically inhibit USP13 induced METTL3 degradation and exhibited significant therapeutic efficacy both *in vitro* and *in vivo*. Collectively, our study results indicate that USP13 promotes glycolysis and tumor progression in OS by stabilizing METTL3, thereby stabilizing ATG5 mRNA and facilitating autophagy in OS. Our findings demonstrate the role of the USP13-METTL3-ATG5 cascade in OS progression and show that USP13 is a crucial DUB for the stabilization of METTL3 and a promising therapeutic target for treating OS.

## Introduction

Osteosarcoma (OS) is a primary bone tumor which originates from mesenchymal cells with a high fatality rate worldwide^1^. Because of limited available therapeutic approaches, the overall survival rate of OS patients with distant metastasis is extremely low^2^. Thus, clarifying the potential mechanisms involved in OS and developing effective and comprehensive approaches to treat OS, remain urgent and important issues.

Rapid cell proliferation and glucose metabolism reprogramming are promoted in tumors. Even under normoxic conditions, tumor cells exhibit promoted glycolysis for rapid energy and biosynthesis production; this phenomenon is known as Warburg effect^3^. Characterizing the underlying mechanisms underlying glycolysis may lead better understandings of tumor progression. Deubiquitinating enzymes (DUBs) are a class of proteases which eliminate the ubiquitination of proteins by cleaving ubiquitin moieties from target proteins^4^, ^5^. So far, over one hundred confirmed DUBs have been identified, divided into several groups such as ubiquitin-specific proteases (USPs), ubiquitin carboxy-terminal hydrolases, and ovarian tumor proteases. Accumulating evidence has demonstrated that DUBs are often dysregulated in various tumors and are critical for regulating cellular homoeostasis and processes which are involved in the development of malignant tumors^6^, ^7^. So far, several DUBs have been studied and proven to be essential in various cancers. However, to the best of our knowledge, no DUBs have been studied in the progression of OS and thus warrants further investigation.

In recent years, epigenetics including DNA methylation, RNA N^6^-methyladenosine (m^6^A), etc. has becoming the new frontier and a hot spot in the field of tumor biology^8^, ^9^. As the most common and abundant post-transcriptional modification of RNAs, m^6^A modification is mainly regulated by m^6^A “writers”, “erasers” and “readers”, and is thought to be associated with RNA translation, stability and decay, thus affecting the fate of RNA^10^-^12^. METTL3, known as a core subunit of the methyltransferase complex, has been reported to be involved in various biological processes including tumor progression, macrophage polarization, axonal growth, atherosclerosis, and chronic hypertrophy among others^13^-^17^. However, the functional role of m^6^A modification, especially the mechanism by which METTL3 mediates the m^6^A modification in OS progression, remains unknown.

In the present study, we identified USP13 as a functional DUB that was upregulated in OS and correlated with poor prognosis. Our results indicated that USP13 promotes glycolytic reprogramming and progression in OS by stabilizing as well as deubiquitinating METTL3 protein at K488 by removing K48-linked ubiquitin chains, which further stabilizes ATG5 mRNA and activates oncogenic autophagy. In addition, we evaluated the potential therapeutic effects of pharmacological inhibition of USP13. Collectively, our data demonstrate the role of the USP13-METTL3-ATG5 cascade in the progression of OS and thus put forward a promising therapeutic target for treating OS.

## Materials and Methods

### Clinical specimens

This present research was approved by the ethics committee of Changzheng Hospital, Second Affiliated Hospital of Naval Medical University. OS and paired adjacent normal specimens were obtained during surgery. Tissue specimens were quickly frozen in liquid nitrogen and stored at -80 °C.

### RNA sequencing (RNA-Seq) and methylated RNA immunoprecipitation sequencing (MeRIP-Seq)

RNA-Seq and MeRIP-Seq were performed and analyzed by Nanjing Jiangbei New Area Biopharmaceutical Public Service Platform Co., Ltd., (Nanjing, China). For RNA-Seq, total RNA was extracted from 143B cells transfected with shMETTL3 or negative control and processed as we previously described^18^. For MeRIP-Seq, total RNAs were extracted from METTL3-knockdown or negative control 143B cells. The extracted mRNA was chemically fragmented into fragments of approximately 100-nt, and immunoprecipitated with anti-m^6^A antibody (Sigma-Aldrich, USA). The eluted m^6^A mRNA fragments were then concentrated to construct an RNA-seq library and sequenced on a NovaSeq 6000 (Illumina Inc., USA). The remainder of the RNAs were used for MeRIP-qPCR to determine the m^6^A level of ATG5 and normalized to the input. Differential expression analysis was performed using a DESeq2 Bioconductor package and adjusted *P* value < 0.05 was set as the cutoff criterion.

### Animal experiments

All animal experiments were carried out approved by the Institutional Animal Care and Use Committee of Naval Medical University. Four-week-old nude mice (BALB/c nude mice) were purchased from the Animal Model Institute of Nanjing University (Nanjing, China) and used for *in vivo* tumor growth and metastasis experiments. For tumor growth assay, 2 × 10^6^ OS cells in 100 μL medium were injected into the nude mice subcutaneously. For tumor metastasis assay, we injected medium containing 2 × 10^6^ cells through the caudal vein. An IVIS200 imaging system (Caliper Life Science, USA) was used to image and assess the OS metastasis. For pharmacological inhibition of USP13, mice bearing xenografts were treated with Spautin-1 (40 mg/kg/day i.p.) or vehicle for 2 weeks.

### Statistical analysis

All data are shown as mean ± standard deviation with at least three independent biological replicates, and statistical analysis was performed in GraphPad Prism (version 8.0, GraphPad Software Inc., USA). The unpaired two-tailed Student's t-test was used for comparisons between two groups, and one-way or two-way ANOVA followed by Tukey's post-hoc test was used for comparisons between more than two groups. P value < 0.05 was considered to be significant statistically.

## Results

### USP13 is upregulated in OS and correlated with a poor outcome

First, we examined USP13 expression in two published datasets obtained from OS samples deposited in GEO (GSE14359 and GSE16088). Data showed that USP13 was markedly upregulated in tumor specimens compared with adjacent normal tissues (Fig. S1a). In addition, data from the Human Cancer Metastasis Database (HCMDB) verified the upregulation of USP13 in OS specimens (Fig. S1b). OS patients with aberrant USP13 expression showed a worse outcome based on an online database (Fig. S1c). Also, results from another online database further confirmed that patients with high USP13 expression in sarcoma had a poorer prognosis (Fig. S1d). Increased USP13 level in OS tissues was verified via immunohistochemistry (IHC) combined with tissue microarray (TMA) (Fig. S1e, f). The upregulation of USP13 in OS tissues was further validated by western blotting in twelve randomly-selected pairs of tumor tissues and corresponding adjacent normal tissues (Fig. S1g). Also, we analyzed USP13 mRNA expression in forty paired tumor specimens and corresponding adjacent normal tissues by qRT-PCR, and our findings further confirmed the upregulation of USP13 mRNA level in OS (Fig. S1h). Moreover, USP13 expression was analyzed in the OS cell lines as well as the normal human osteoblast cell line using western blotting. Consistently, the expression level of USP13 was shown to be increased in OS cell lines (Fig. S1i). In all, these data showed that USP13 is involved in OS progression and associated with poor prognosis.

### USP13 promotes glycolysis and cell proliferation in OS

**Figure 1:**
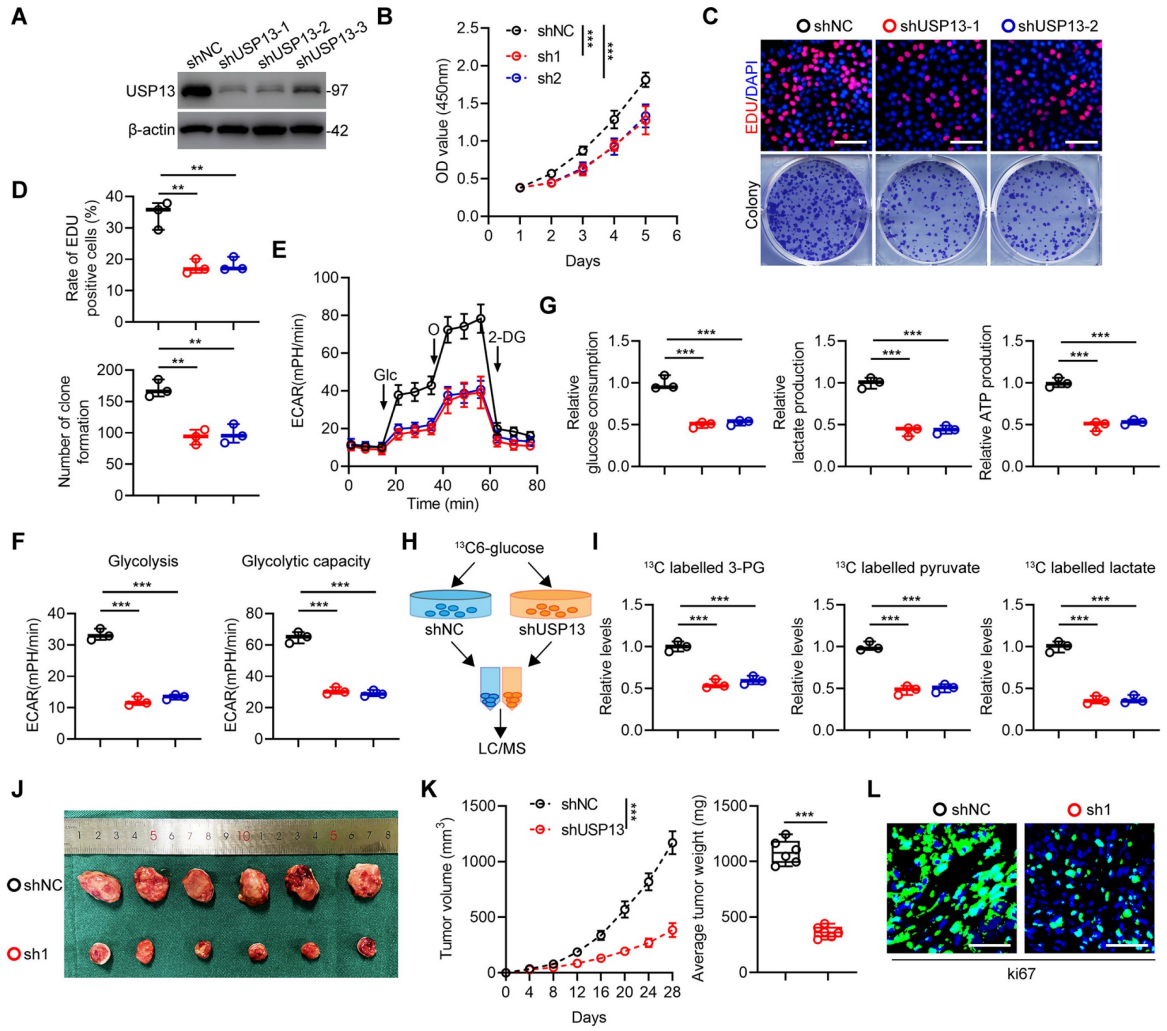
USP13 promotes glycolysis and cell proliferation in OS. (a) USP13 expression levels after transfection of shUSP13 into 143B cells were detected by western blot analysis. (b) CCK-8 assays in 143B cells with USP13 depletion. (c, d) Representative images and quantifications of mitotic cells and colony number of 143B cells with USP13 depletion. Scale bar = 200μm. (e) Extracellular acidification rate (ECAR) was measured in 143B cells with USP13 depletion. (f) Quantification of glycolysis and glycolytic capacity. (g) Glucose consumption, lactate production and ATP production were measured in 143B cells with USP13 depletion. (h) Flowchart of glucose metabolic flux analysis. (i) ^13^C-labeled metabolic intermediates of glycolysis were measured in 143B cells with USP13 depletion. (j) Representative images of xenograft tumors in USP13-depleted and negative control 143B cells groups. (k) The growth and weight of xenografts tumors in USP13-depleted and negative control 143B cells groups. (l) Ki67 staining in indicated groups. Scale bar = 50μm.

Due to the high expression level of USP13 in 143B cells, 143B cells were chosen to silence USP13. It was shown that sh-1 and sh-2 have highest knockdown efficiency, thus were selected for following research (Fig. 1a). Moreover, low USP13-expressing HOS cells were chosen to overexpress USP13 stably (Fig. S2a). According to data from CCK-8 assays, cell proliferation ability was significantly inhibited after depleting USP13, but promoted after overexpression of USP13 in OS cells (Fig. 1b, S2b). Also, EdU as well as colony-formation assays further confirmed downregulating USP13 suppressed cell proliferation whereas upregulation of USP13 significantly promoted cell proliferation in OS cells (Fig. 1c, d and S2c, d). Then, we explored the effects of USP13 on aerobic glycolysis and found that silencing USP13 in OS cells decreased levels of extracellular acidification rate (ECAR, Fig. 1e) and reduced glycolysis and glycolytic capacity (Fig. 1f). Also, glucose consumption, lactate production as well as cellular ATP levels were reduced in USP13-downregulated OS cells (Fig. 1g). Further, the intracellular amounts of ^13^C-labeled metabolic intermediates in OS cells after coculturing with ^13^C-glucose were examined to investigate the metabolic flux (Fig. 1h). Based on liquid chromatography and mass spectrometry (LC/MS), the metabolome analysis indicated that intracellular metabolites of glycolysis including 3-phosphoglycerate, pyruvate and lactate were evidently declined after USP13 depletion (Fig. 1i), further verifying that USP13 is crucial for glycolysis process. In contrast, overexpression of USP13 in OS cells showed promoted glycolysis (Fig. S2e-i).

Next, we continued to investigate the role of USP13 in OS progression *in vivo* and injected transfected 143B and HOS cells into nude mice subcutaneously. As presented in Fig. 1j, k, the volume and weight of tumors in USP13 depletion group were obviously decreased compared with those in control group. Moreover, Ki67 staining showed a reduced proliferation rate in USP13-knockdown xenografts (Fig. 1l). Conversely, overexpression of USP13 promoted xenografted tumor growth, size and weight *in vivo* (Fig. S2j, k). In addition, Ki67 staining also showed an enhanced proliferation ratio (Fig. S2l). In all, these findings showed that USP13 acts a key role in promoting OS proliferation and glycolysis both *in vitro* and *in vivo*.

### USP13 promotes OS cells metastasis

**Figure 2:**
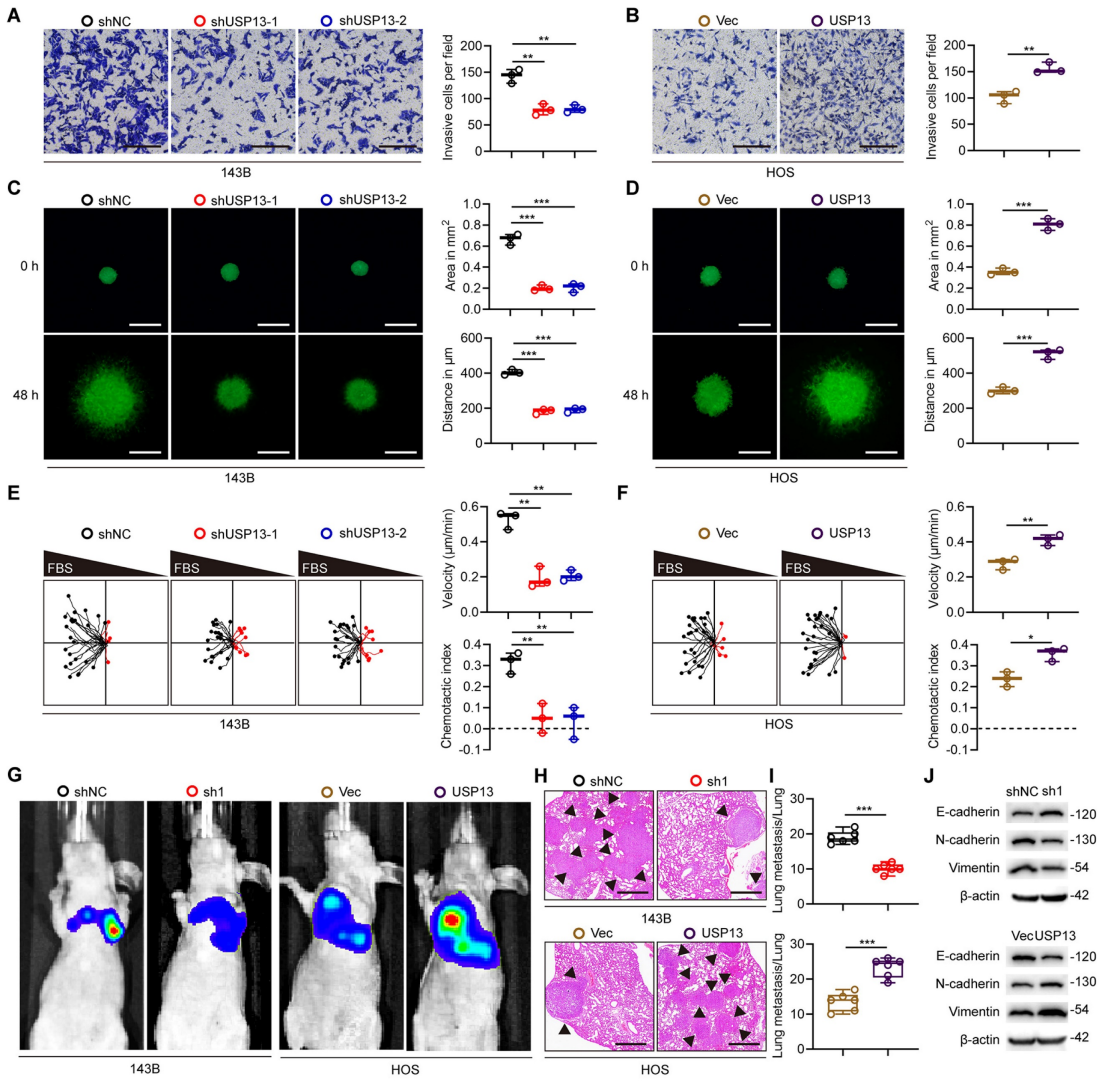
USP13 promotes OS cells metastasis. (a, b) Representative images and quantifications of invasion of OS cells in indicated groups. Scale bar = 200μm. (c, d) Representative images and quantifications of 3D tumor spheroid cell-invasion assay in indicated groups. Scale bar = 500μm. (e, f) Migration plots and quantifications of OS cells in a gradient of FBS in indicated groups. The starting site of each trajectory was normalized to the positions x = 0 and y = 0. The average velocities and chemotaxis indices of OS cells were evaluated. (g) Representative images of pulmonary metastasis after injection of 143B and HOS cells via tail vein in indicated groups. (h, i) Representative images and quantification of H&E staining of pulmonary metastatic nodules. Scale bar = 500μm. (j) Expression levels of EMT-related proteins in indicated xenografted tumors.

Various experiments were conducted to further explore the role of USP13 in OS cells metastasis. Downregulation of USP13 significantly attenuated the invasiveness of 143B cells, while overexpression of USP13 promoted the invasiveness of HOS cells by using the transwell invasion assay (Fig. 2a, b). We further demonstrated that USP13 obviously promoted OS cells invasion using a 3D tumor spheroid cell-invasion assay (Fig. 2c, d). Additionally, the migration effect of USP13 on OS cells was explored using a microfluidic migration chamber with stable, shear-minimized linear chemo-attractive gradients as we previously described^18^. Silence of USP13 markedly inhibited the migration and directionality of 143B cells to FBS compared with the control (Fig. 2e). However, USP13-upregulated HOS cells were more sensitive to the FBS gradient and exhibited a stronger directed migration (Fig. 2f).

Further, stably-transfected OS cells were injected into tail vein of nude mice to evaluate the role of USP13 in OS cells metastasis *in vivo*. Pulmonary metastasis was significantly suppressed in USP13-depletion group, but obviously promoted in USP13-upregulation group after six weeks (Fig. 2g). Then the mice were sacrificed and pulmonary sections were obtained and evaluated. Results from hematoxylin and eosin (H&E) staining in indicated groups further confirmed above results (Fig. 2h, i). Epithelial-mesenchymal transition (EMT) plays an important role in the development of malignant tumors and we further evaluated the expression of proteins associated with EMT. As shown in Fig. 2j, the expression levels of mesenchymal markers including N-cadherin and Vimentin was decreased while epithelial cell marker E-cadherin was upregulated in USP13-depletion xenografted tumors. In contrast, the opposite effect was found in USP13-upregulation xenografted tumors. Taken together, these data indicated that USP13 accelerated OS metastasis *in vitro* and *in vivo*.

### USP13 interacts with and stabilizes METTL3

**Figure 3:**
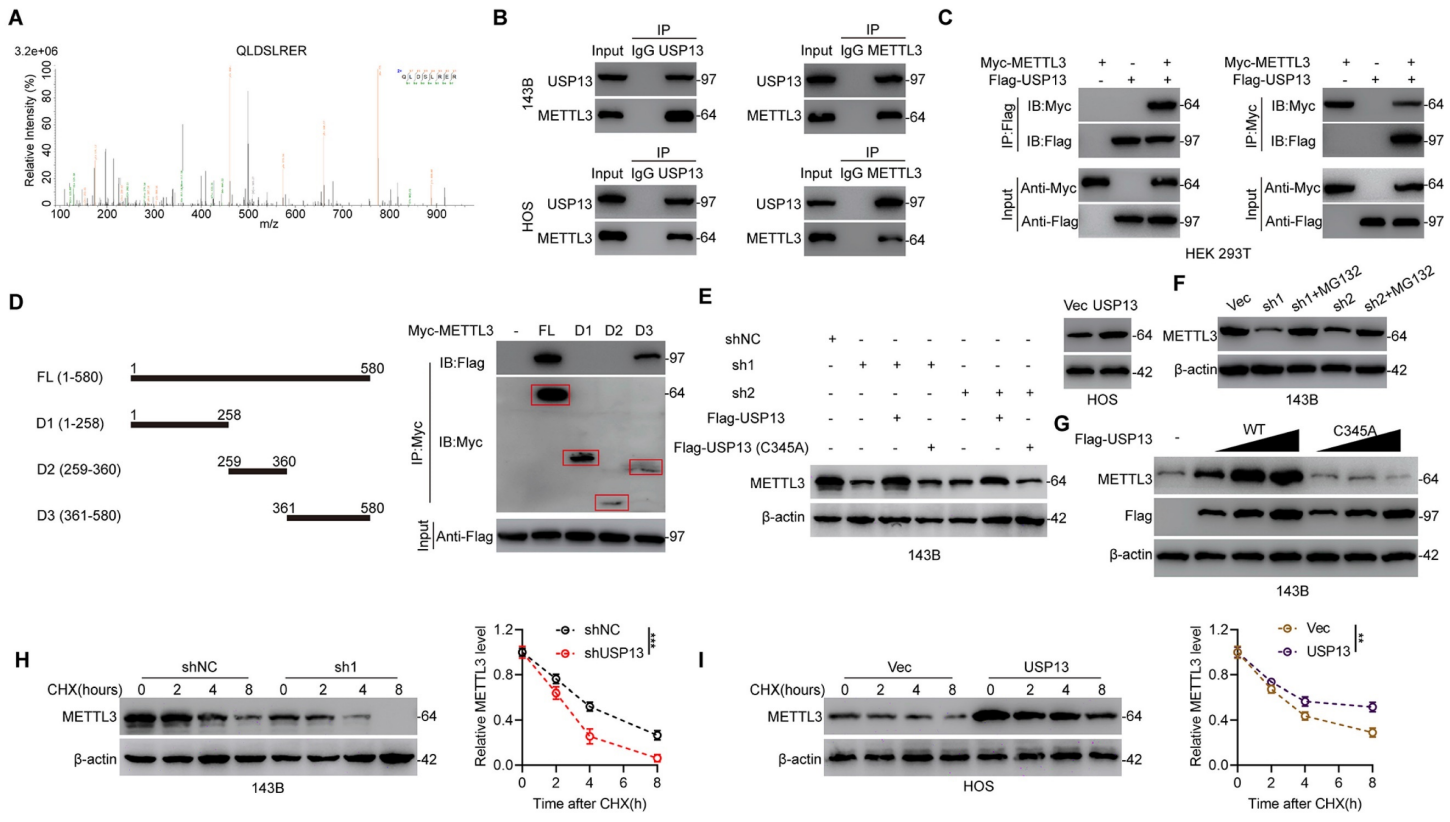
USP13 interacts with and stabilizes METTL3. (a) IP/MS analysis indicated METTL3 as a USP13-interacting protein. (b) Cell lysates of OS cells were immunoprecipitated with USP13 and METTL3 antibodies respectively and subsequently immunoblotted using METTL3 and USP13 antibodies respectively to detect endogenous protein interactions. (c) Cell lysates of HEK 293T cells were transfected with Flag-tagged USP13 and Myc-tagged METTL3 plasmids and immunoprecipitated with Flag and Myc antibodies respectively and immunoblotted using Myc and Flag antibodies respectively to detect exogenous protein interactions. (d) Schematic representation of Myc-tagged full-length (FL) METTL3 and various deletion fragments. HEK 293T cells were co-transfected with Flag-USP13 and Myc-tagged METTL3 FL or its deletion mutants, and cell lysates were examined by immunoprecipitation followed by immunoblotting with anti-Flag and anti-Myc. (e) METTL3 protein levels in indicated groups. (f) METTL3 protein expression in USP13-depleted OS cells with or without treating MG132. (g) METTL3 and Flag-USP13 protein levels in 143B cells with increasing expression of Flag-USP13 (WT or C345A mutant). (h, i) METTL3 protein levels with cycloheximide (CHX, 10 μg/mL) treatment in indicated groups.

Further, to investigate the underlying mechanism in USP13-regulated OS glycolysis and progression, IP coupled with MS (IP/MS) was performed to explore which protein binds to USP13. Interestingly, the m^6^A writer METTL3 was identified as a protein binding to USP13 (Fig. 3a). Co-immunoprecipitation (Co-IP) analysis showed that endogenous METTL3 was present in endogenous USP13 immunoprecipitates from OS cells and reverse Co-IP verified that USP13 could be precipitated by METTL3 as well (Fig. 3b). Moreover, the interaction between USP13 and METTL3 was verified by Co-IP analysis in HEK 293T cells with exogenously transfection of Flag-tagged USP13 and Myc-tagged METTL3 (Fig. 3c). Using full-length and a series of truncated Myc-tagged METTL3 fragments to examine potential binding areas in HEK 293T cells, our findings suggested that fragments including amino acids 361-580 of METTL3 could interact with USP13 (Fig. 3d). Taken together, our results confirm that USP13 interacts with METTL3.

Since USP13 binds to METTL3, we then explored the effect of downregulating or upregulating USP13 on METTL3 stability in OS cells. Knockdown or overexpression of USP13 markedly decreased or increased METTL3 protein but not mRNA levels (Fig. 3e, S3). In addition, knockdown of USP13 significantly decreased the protein level of METTL3, which could be reversed by WT but not catalytically inactive C345A mutant USP13 (Fig. 3e). Interestingly, addition of MG132 which is a proteasome inhibitor abolished the downregulation of METTL3 protein after knockdown of USP13 (Fig. 3f). Further analysis was performed to investigate if METTL3 can be stabilized by USP13. METTL3 protein level was significantly increased after overexpressing USP13, while not affected by overexpressing catalytically inactive C345A mutant USP13 (Fig. 3g). Further, we continued to examine the influence of overexpression or depletion of USP13 on the endogenous METTL3 protein stability in the presence of cycloheximide (CHX), an inhibitor of protein synthesis inhibitor. Silencing of USP13 significantly accelerated METTL3 degradation while upregulation of USP13 obviously inhibited METTL3 degradation (Fig. 3h, i).

### USP13 deubiquitinates METTL3 at K488 by removing K48-linked ubiquitin chains

**Figure 4:**
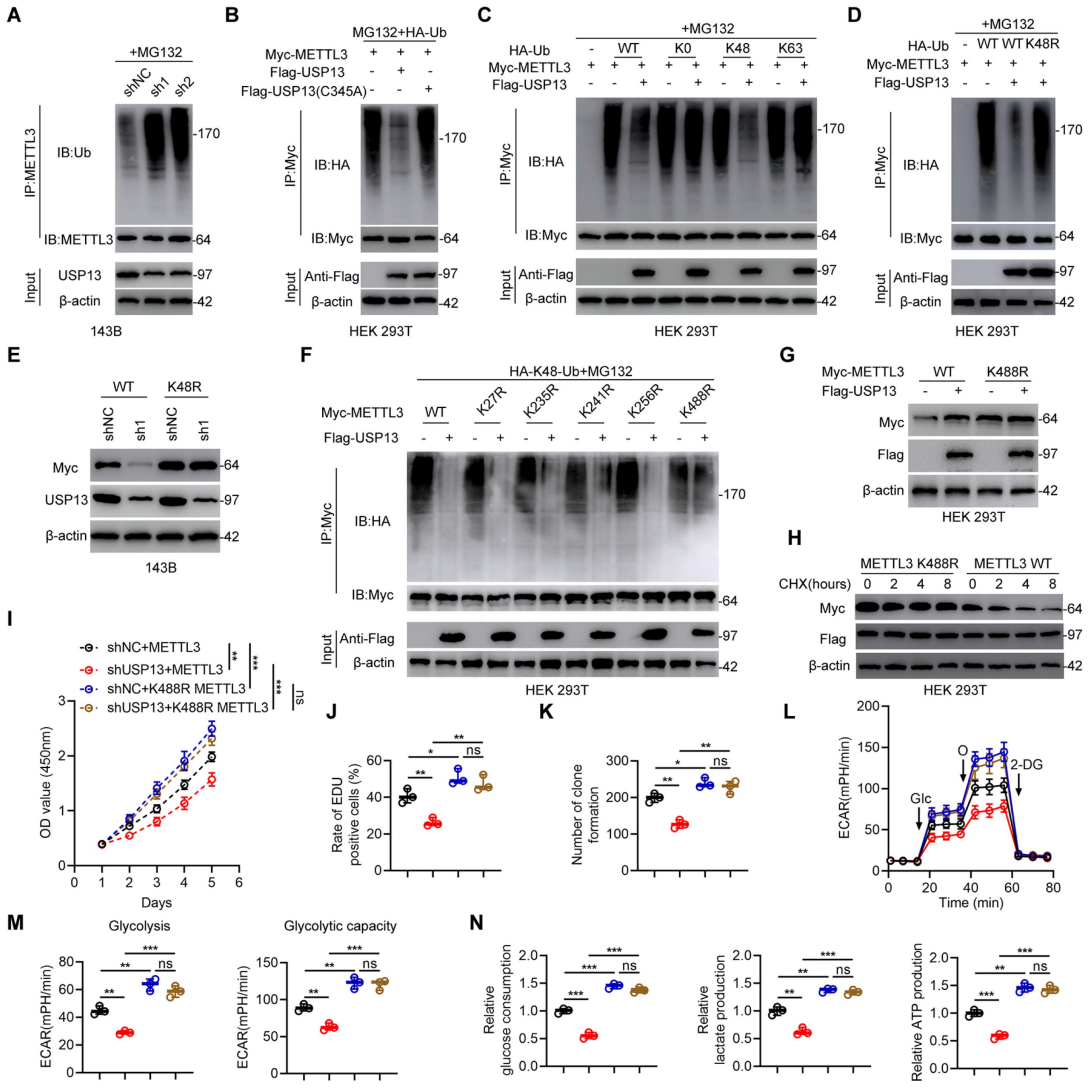
USP13 deubiquitinates METTL3 at K488 by removing K48-linked ubiquitin chains. (a) Endogenous METTL3 ubiquitination level in 143B cells in shNC and shUSP13 groups. (b) Exogeneous METTL3 ubiquitination level in HEK 293T cells co-transfected with Flag-tagged USP13 (WT or C345A), HA-tagged Ub and Myc-tagged METTL3. (c) Evaluation of METTL3 ubiquitylation linkage in HEK 293T cells co-transfected with Flag-USP13, Myc-METTL3 and the specific HA-Ub, K0, K48-only, or K63-only plasmids. (d) Evaluation of METTL3 ubiquitylation linkage in HEK 293T cells were co-transfected with Flag-USP13, Myc-METTL3 and the specific WT and K48R HA-Ub plasmids. (e) Evaluation of Myc-METTL3 and USP13 protein expression levels in 143B cells transfected with WT and K48R HA-Ub as well as Myc-METTL3 in the presence of shNC or shUSP13. (f) Crucial lysine site of METTL3 deubiquitinated by USP13 were evaluated by co-transfecting Flag-USP13, HA-Ub, Myc-METTL3 WT and mutants in HEK 293T cells. (g) Evaluation of Myc-METTL3 and Flag-USP13 protein expression levels in HEK 293T cells transfected with WT and K488R Myc-METTL3 in the presence of Flag-Vec or Flag-USP13. (h) Detection of Myc-METTL3 protein levels in HEK 293T cells co-transfected with WT or K488R METTL3, and Flag-USP13 in the presence of CHX (10 μg/mL) for the indicated times. (i-n) Quantification of CCK-8 assays (i), EdU assays (j), colony formation assays (k), ECAR (l), glycolysis and glycolytic capacity (m), glucose consumption, lactate production and ATP production (n) in 143B cells in indicated groups.

Since USP13 is a DUB and stabilizes METTL3, relevant analyses were performed to explore whether USP13 regulates METTL3 ubiquitination and degradation. Depletion of USP13 significantly promoted METTL3 ubiquitination level compared with shNC in 143B cells (Fig. 4a). In contrast, overexpression of USP13 significantly decreased ubiquitination of METTL3 in HOS cells (Fig. S4a). In addition, METTL3 ubiquitination level was reduced in tumor tissues which highly expressed USP13 compared to adjacent normal tissues (Fig. S4b). Tumors from *in vivo* USP13 depletion group showed upregulated METTL3 ubiquitination level compared to tumors from control group (Fig. S4c). To further confirm the role of USP13 on METTL3 ubiquitination, HEK 293T cells were co-transfected with Flag-USP13 (either WT or C345A mutant), Myc-METTL3 and HA-Ub. Overexpression of WT USP13 decreased METTL3 ubiquitination while C345A mutant USP13 showed no significant effect (Fig. 4b). It's generally recognized that K48- and K63-linked chains are two main types of polyubiquitin chain, and our results showed that USP13 cleaved K48-linked polyubiquitin chains from METTL3 instead of K63-linked polyubiquitin chains (Fig. 4c). Next, we mutated K48 ubiquitination site and found that USP13-mediated deubiquitination of METTL3 was abolished (Fig. 4d). To further confirm that K48-linked polyubiquitination is essential for USP13-reguated METTL3 protein stabilization, we transfected K48R type of ubiquitin and Myc-METTL3 into USP13-depletion 143B cells and found that transfection of K48R ubiquitin reversed the decrease in Myc-METTL3 protein level in USP13-depletion cells (Fig. 4e). In all, these findings indicate that USP13 regulates METTL3 stability by removing K48-linked ubiquitin chains in OS cells.

Next, to investigate the crucial lysine site responsible for USP13-regulated METTL3 deubiquitination, we mutated five lysine residues to arginine individually in METTL3 according to a previous study^19^. Our results indicated that mutation of K488 in METTL3 obviously abolished the reduced METTL3 ubiquitination level when overexpression of USP13, while mutation of the other 4 lysine sites exhibit no influence (Fig. 4f). Further, as shown in Fig. 4g, the ectopic expression of USP13 led to an increase of WT but not the K488R mutant of METTL3. Consistently, the half-life of the METTL3 K488R mutant was increased than that of WT METTL3 (Fig. 4h). Accordingly, these results suggest that K488 of METTL3 is the major deubiquitination target residue of USP13.

Further, to study the key role of the ubiquitination site K488 of METTL3, we silenced endogenous USP13 in 143B cells and then ectopically expressed WT and K488R of METTL3. We divided this part of experiments into four groups: shNC+METTL3; shUSP13+METTL3; shNC+K488R METTL3; shUSP13+K488R METTL3. Ectopically expressed K488R of METTL3 significantly promoted cell proliferation and glycolysis, while USP13 knockdown decreased cell proliferation and glycolysis with WT METTL3 rather than K488R METTL3, which indicated that mutation of K488 of METTL3 promoted cell proliferation and glycolysis and abolished USP13 depletion-induced proliferation and glycolysis suppression (Fig. 4i-n). In all, these results demonstrated that USP13 regulates glycolytic reprogramming and proliferation in OS via the deubiquitination of METTL3 at K488.

### METTL3 is upregulated and positively correlated with USP13 in OS

Since METTL3 is a substrate of USP13, we next investigated the correlation between the two proteins. Online database results showed that METTL3 was upregulated in OS specimens (Fig. S5a). Kaplan-Meier analysis further indicated that OS patients with a high METTL3 level had a worse clinical outcome (Fig. S5b). The expression level of METTL3 mRNA was examined in forty paired tumor specimens and corresponding adjacent normal tissues, and results confirmed the upregulation of METTL3 mRNA level in OS tissues (Fig. S5c). Further, METTL3 protein expression level was evaluated in twelve random pairs of OS tissues as well as adjacent normal tissues by western blotting and verified the upregulation of METTL3 protein level in OS tissues (Fig. S5d). Furthermore, using Spearman correlation test, we found a marked positive correlation between the protein expression of USP13 and METTL3 (Fig. S5e). Moreover, METTL3 level was shown to be upregulated in OS cell lines (Fig. S5f). Our results showed that METTL3 is upregulated and positively correlated with USP13 in OS.

### USP13 promotes glycolysis and progression in OS by binding to and stabilizing METTL3

Next, we investigated the role of METTL3 in the mechanism by which USP13 promotes glycolysis and progression of OS cells via lentiviral-mediated METTL3 overexpression in USP13-silenced 143B cells and METTL3 silencing in USP13-upregulated HOS cells. A series of *in vitro* rescue experiments as described above were then carried out. It was found that the decreased glycolysis, cell proliferation, migration and invasion in USP13-depletion 143B cells were abolished after overexpressing METTL3, whereas METTL3 silencing diminished the promoted glycolysis and malignant phenotypes in USP13-overexpression HOS cells (Fig. S6).

**Figure 5:**
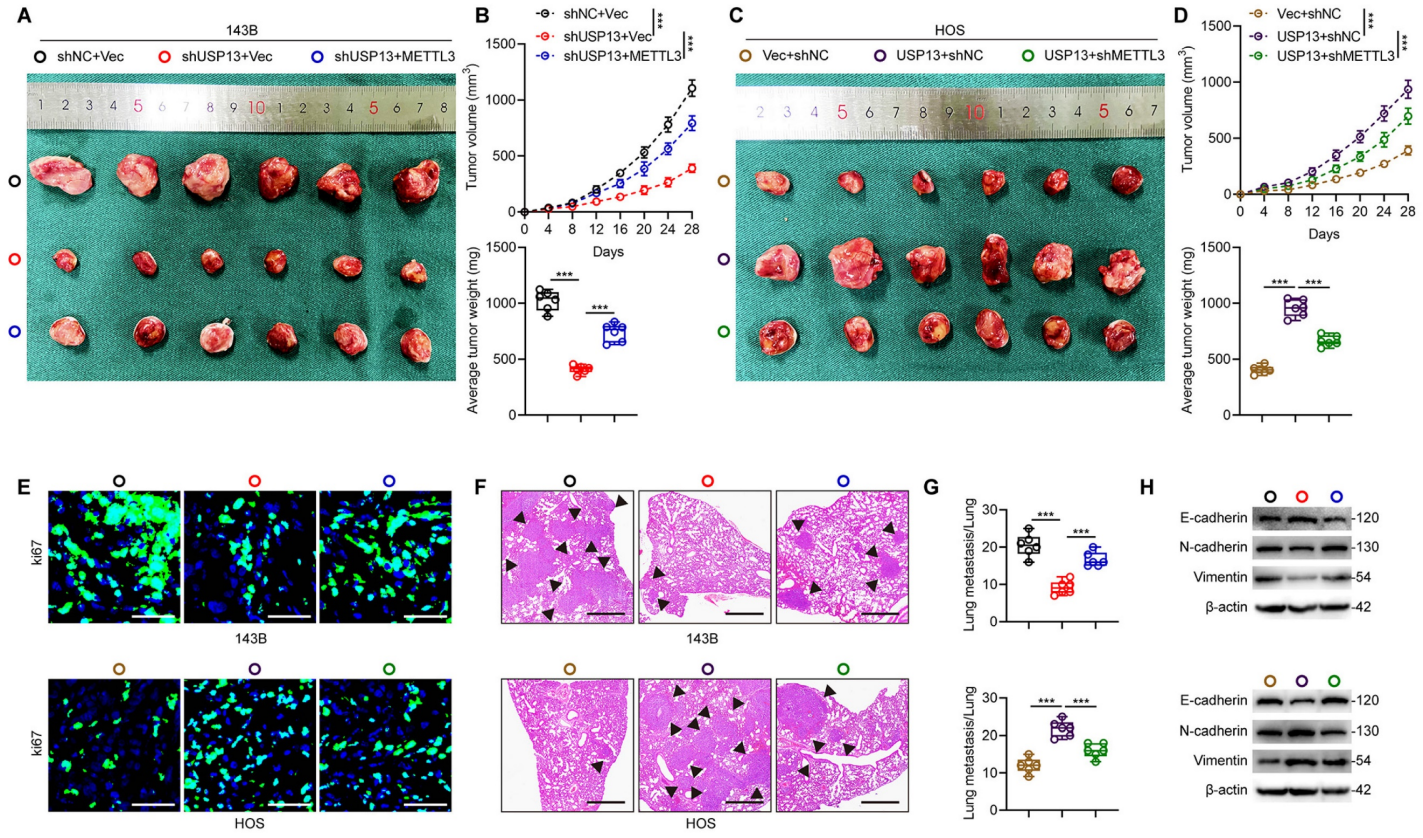
USP13 promotes OS malignancy by binding to and stabilizing METTL3 *in vivo*. (a, c) Representative images of tumors in nude mice in the indicated groups. (b, d) Quantification of tumor volume and weight in nude mice in the indicated groups. (e) IF staining of Ki67 in tumor sections from the indicated groups. Scale bar = 50μm. (f, g) Representative images and quantification of H&E staining of pulmonary metastatic nodules in indicated groups. Scale bar = 500μm. (h) Expression levels of EMT-related proteins in indicated xenografted tumors.

Next, we generated xenografts in nude mice to explore whether METTL3 is a crucial downstream target for USP13, which promotes OS progression. The reduced volume and weight of tumor nodules caused by USP13 silence was found to be counteracted by ectopic overexpression of METTL3 in 143B cells (Fig. 5a, b). Conversely, depletion of METTL3 in HOS cells reversed USP13-mediated OS progression (Fig. 5c, d). Images from Ki67 staining confirmed these results (Fig. 5e). In pulmonary metastatic models, a reduction in pulmonary metastasis caused by USP13 knockdown was abolished in 143B cells with METTL3 overexpression, while silencing METTL3 in HOS cells markedly counteracted the pro-metastatic influence of USP13 upregulation on pulmonary metastasis, as evidenced by histological analysis (Fig. 5f, g). Moreover, the suppressed EMT in USP13-depletion xenografted tumors was found to be counteracted by ectopic expression of METTL3 while the promoted EMT in USP13-upregualtion xenografted tumors was abolished by silencing METTL3 (Fig. 5h). Collectively, these findings demonstrated that METTL3 is the downstream target of USP13 and stabilization of METTL3 is essential for the role of USP13 in OS malignant progression.

### ATG5 is identified as an oncogene of the USP13/METTL3 axis

**Figure 6:**
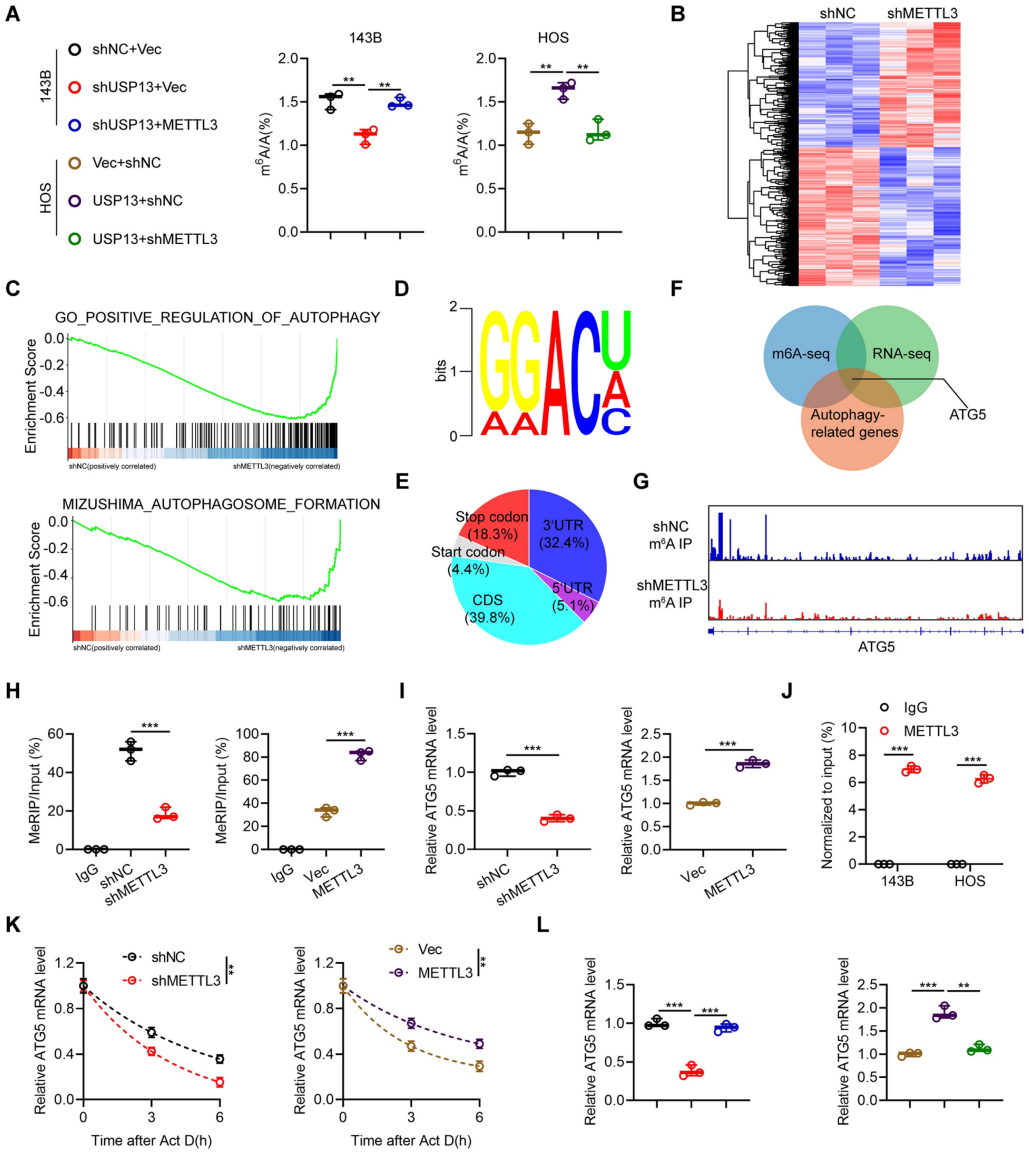
Regulation of m^6^A-modified ATG5 mRNA stability by the USP13/METTL3 axis. (a) The global mRNA m^6^A modification in OS cells in indicated groups was performed and quantified by LC-MS/MS. (b) Heat maps of DEGs in 143B cells with or without METTL3 knockdown; red and blue colors represent high and low expression values, respectively. (c) Gene set enrichment analysis (GSEA) was used to identify the distribution of genes in the autophagy-related pathway gene set of 143B cells transfected with shNC or shMETTL3. (d) The m^6^A consensus sequence motif was identified in 143B cells. (e) Distribution of m^6^A modification in mRNA transcripts within different gene regions. (f) Overlapping analysis of autophagy-related genes identified by RNA-Seq and MeRIP-Seq. (g) m^6^A peaks at ATG5 mRNA was visualized through integrative genomics viewer (IGV). (h) MeRIP-qPCR of the transcript of ATG5 in OS cells in indicated groups. (i) ATG5 mRNA expression levels in OS cells in indicated groups. (j) METTL3 RIP assays of ATG5 transcripts in METTL3-bound mRNAs in OS cells. (k) RNA lifetime of ATG5 in the indicated OS cells after transcription inhibition (actinomycin D, 5 μg/mL). (l) ATG5 mRNA expression level in OS cells of indicated groups.

Since METTL3 is a well-recognized m^6^A writer and plays various important roles in m^6^A-modifed transcripts, we further investigated whether USP13 regulates cellular m^6^A abundance by stabilizing METTL3. We used an LC-MS/MS assay to evaluate cellular m^6^A enrichment and found that silencing of USP13 markedly decreased the m^6^A level while this downregulation of m^6^A level was significantly rescued by overexpressing METTL3 in 143B cells. Meanwhile, overexpression of USP13 resulted in a significant upregulation of m^6^A abundance while this increase was abolished by depletion of METTL3 in HOS cells (Fig. 6a). These results supported that USP13-mediated regulation of m^6^A abundance is dependent on METTL3.

Next, we conducted transcriptome analysis by RNA-Seq. As shown in Fig. 6b, silencing of METTL3 led to 617 genes upregulation and 687 genes downregulation in 143B cells. Further, gene set enrichment analysis (GSEA) was conducted and indicated METTL3 was remarkedly involved in autophagy pathway, indicating a possible functional effect of METTL3 on autophagy regulation (Fig. 6c). Further, MeRIP-seq was performed to investigate the potential target mRNA of METTL3 in 143B cells. The m^6^A consensus sequence identified in 143B cells was GGAC, suggesting the successful enrichment of m^6^A-modified mRNAs (Fig. 6d). Additionally, we found that these m^6^A modification sites were mostly located in coding regions and 3'-untranslated regions (Fig. 6e). By overlapping the results from methylated RNA immunoprecipitation sequencing (MeRIP-Seq), RNA-Seq and identification of autophagy-related genes, ATG5 was ultimately identified as the potential target of METTL3 (Fig. 6f). Integrative genomics viewer (IGV) plots of m^6^A peaks in ATG5 mRNA, identified using an m^6^A-specific antibody, are shown in Fig. 6g. MeRIP-qPCR analyses indicated that the m^6^A modifications of ATG5 were decreased by downregulation of METTL3 in 143B cells and increased by upregulation of METTL3 in HOS cells (Fig. 6h). Further, we demonstrated that silencing of METTL3 significantly decreased ATG5 mRNA expression in 143B cells while overexpression of METTL3 increased ATG5 mRNA expression in HOS cells (Fig. 6i). Moreover, the interactions between METTL3 and ATG5 mRNA in both 143B and HOS cells were confirmed by RIP-qPCR analysis using a METTL3-specific antibody (Fig. 6j). Next, we speculated that the stability of ATG5 mRNA might be influenced by m^6^A modification. The half-life of ATG5 mRNA was significantly decreased by silencing of METTL3 in 143B cells but upregulated by overexpressing METTL3 in HOS cells after pretreatment with actinomycin D (Fig. 6k). Also, our results indicated that upregulation of METTL3 in USP13-knockdown 143B cells rescued the downregulated ATG5 mRNA, while silencing of METTL3 in USP13-overexpressing HOS cells decreased the upregulated ATG5 mRNA level (Fig. 6l). Furthermore, our findings demonstrated that ATG5 expression is positively correlated with both USP13 expression and METTL3 expression in OS tissues (Fig. S7). Thus, we concluded that USP13-mediated regulation of ATG5 is dependent on METTL3.

Indeed, methylated mRNAs are subsequently recognized and bound by reader proteins, to investigate which reader protein is responsible for regulation of methylated ATG5 transcript, we first calculated the correlation coefficient between ATG5 and all reader genes based on datasets of GSE14359 and GSE16088. We found that the IGF2BP3, a member of IGF2BP family which generally maintains mRNA stability and is significantly upregulated in OS tissues, was positively correlated with ATG5 (Fig. S8a-c). The RIP assay further verified the association between ATG5 transcript and IGF2BP3 protein (Fig. S8d). Additionally, knockdown of IGF2BP3 significantly reduced ATG5 level and attenuated its mRNA stability (Fig. S8e-f). Taken together, these findings demonstrated that USP13 upregulated ATG5 via an m^6^A-dependent mechanism through binding with and stabilizing the m^6^A writer METTL3 in OS.

### USP13 promotes oncogenic autophagy dependent on METTL3-mediated upregulation of ATG5 in OS cells

**Figure 7:**
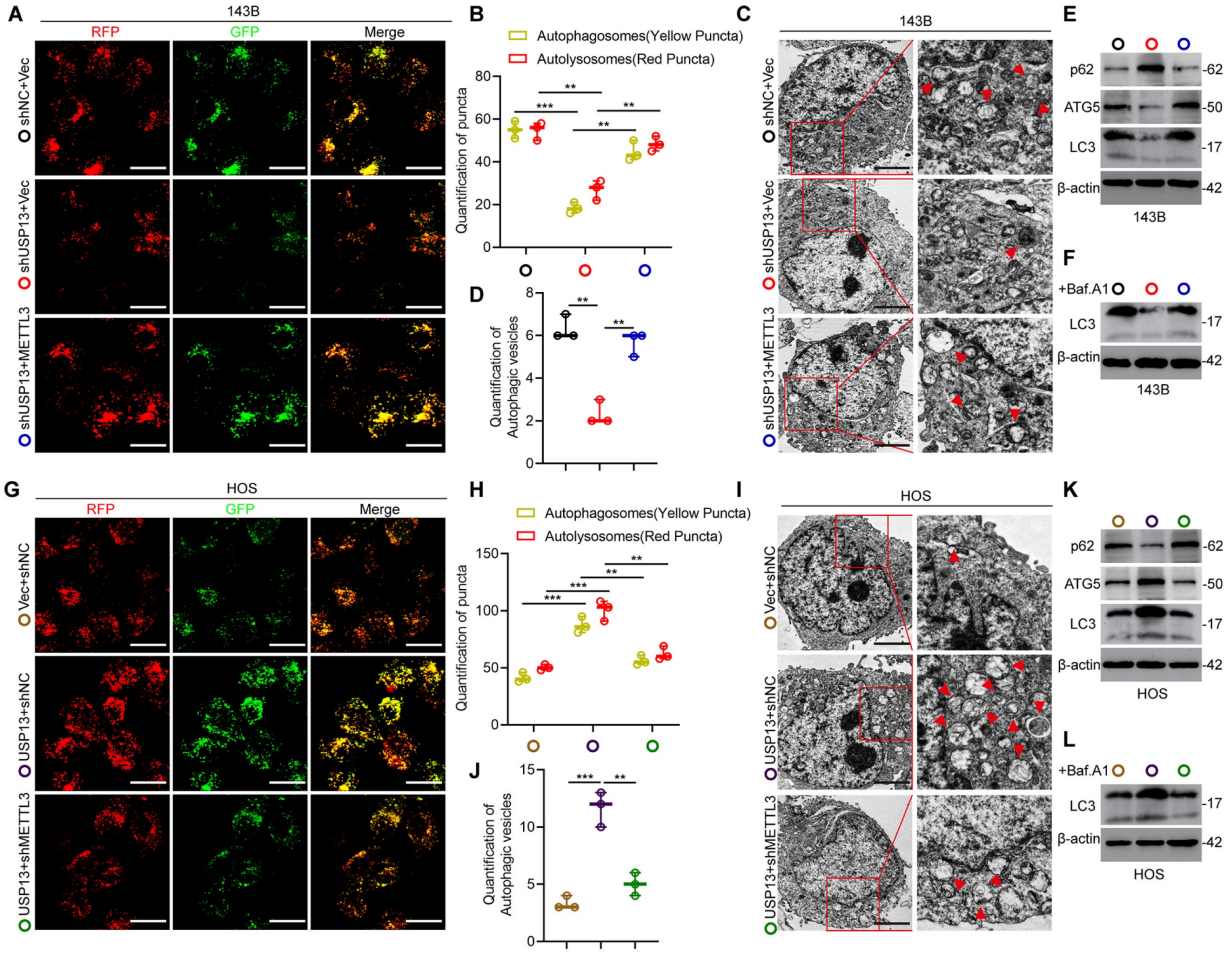
USP13 promotes autophagy dependent on METTL3-mediated upregulation of ATG5 in OS cells. (a, b, g, h) OS cells labelled with GFP-mRFP-LC3 lentivirus were detected, and the cellular puncta were determined in indicated groups. Rescue experiments of USP13 knockdown were performed via ectopic overexpression of METTL3 in 143B cells. Rescue experiments of USP13 overexpression were conducted by downregulating METTL3 in HOS cells. Scale bar = 20μm. (c, d, i, j) TEM was used to assess the autophagic microstructure of 143B and HOS cells in indicated groups. Scale bar = 2μm. (e, f, k, l) Western blot of ATG5, LC3 and p62 in transfected 143B and HOS cells in the presence or absence of the lysosomal inhibitor bafilomycin A1 (Baf.A1).

Considering that ATG5 is a critical gene associated with autophagosome elongation and has been reported to be an oncogene in the progression of OS^20^, ^21^, we therefore determined the functional role of the USP13/METTL3 axis in regulating autophagy. Firstly, OS cells were transfected with GFP-mRFP-LC3 to examine the autophagy flux. Knockdown of USP13 resulted in a great decrease in the number of both non-acidic (mCherry+GFP+) and acidic (mCherry+GFP-) punctate, indicating that the autophagosomes formation and autophagy flux is significantly inhibited, while this effect was greatly reversed by METTL3 overexpression (Fig. 7a, b). Also, transmission electron microscopy (TEM) was used to detect autophagosomes and the results confirmed the above results (Fig. 7c, d). Moreover, analyzing LC3 levels in the presence or absence of the lysosomal inhibitor bafilomycin A1 (Baf.A1) revealed that autophagy flux is impaired when depletion of USP13, while overexpression of METTL3 partially reversed the inhibition (Fig. 7e, f). It's known to us that p62, as another autophagy indicator, is an autophagosome cargo protein which degrades in autolysosomes. Our results indicated that knockdown of USP13 resulted in upregulation of p62, while overexpression of METTL3 partially abolish the increase (Fig. 7e). Taken together, overexpression of METTL3 significantly recovered the formation of autophagosome and inhibition of autophagy flux caused by depletion of USP13 (Fig. 7a-f).

To further study the functional role of METTL3 in regulation of autophagy by USP13, we silenced METTL3 and investigated whether the upregulation of autophagy flux by USP13 overexpression was abolished. It was found that knockdown of METTL3 reversed the formation of autophagosomes and autolysosomes in USP13-overexpression cells (Fig. 7g, h). Also, results from TEM also confirmed the above results (Fig. 7i, j). In addition, our data showed that the increase of LC3 levels and degradation of p62 promoted by USP13 were significantly compromised when knocking down METTL3 (Fig. 7k, l). Collectively, depletion of METTL3 inhibited the formation of autophagosome and autolysosome promoted by ectopic expression of USP13 (Fig. 7g-l). In all, these results indicated that USP13 promoted autophagy dependent on METTL3 in OS cells (Fig. 7).

To investigate whether ATG5-mediated autophagy in OS cells is dependent on its m6A modification, we then constructed a mutant for the m^6^A consensus sequence of ATG5 and transfected it into HOS cells. We found that overexpressing wide type ATG5 significantly facilitated HOS cells autophagy and increased LC3 level, whereas overexpressing mutated ATG5 was comparable with vector control group (Fig. S9).

### Pharmacological inhibition of USP13 weakens the progression of OS cells

**Figure 8:**
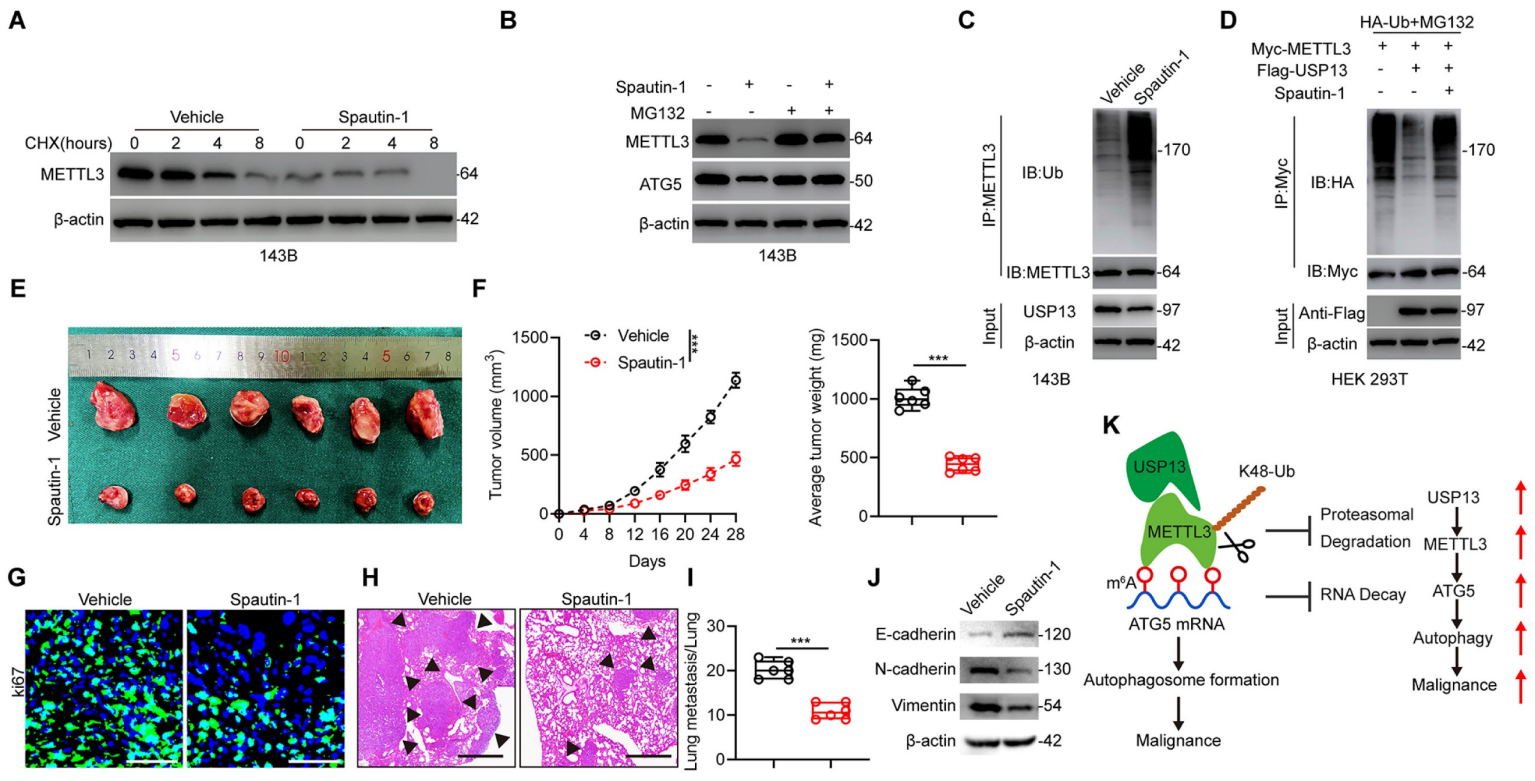
Pharmacological inhibition of USP13 weakens the progression and malignancy of OS cells. (a) 143B cells were treated with 10 μM Spautin-1 or vehicle as well as 10 μg/mL CHX and subsequently subjected to western blot for METTL3 level detection. (b) Western blot analysis of METTL3 and ATG5 in 143B cells treated with Spautin-1 or vehicle with or without MG132. (c) Lysates from 143B cells treated with Spautin-1 or vehicle were subjected to IP with METTL3 antibody followed by IB with indicated antibodies. (d) HEK 293T cells were co-transfected with HA-Ub, Flag-USP13 and Myc-METTL3 in the absence or presence of Spautin-1, and cell lysates were subjected to IP with Myc followed by IB with indicated antibodies. (e) Representative images of xenograft tumors after treatment with Spautin-1 (40 mg/kg/day i.p.) or vehicle for 2 weeks. (f) Quantification of volume and weight of tumor nodules in indicated groups. (g) IF staining of Ki67 in tumor sections. Scale bar = 50μm. (h, i) Representative images and quantification of H&E staining of pulmonary metastatic nodules. Scale bar = 500μm. (j) Expression levels of EMT-related proteins in indicated xenografted tumors. (k) Potential underlying mechanism that USP13/METTL3 axis regulates the m^6^A-dependent ATG5 mRNA.

In order to apply our findings to the clinical situation, we further evaluated the potential pharmacologic inhibitory effect of USP13. A small-molecule inhibitor of USP13 named Spautin-1 was introduced to evaluate whether the deubiquitinating ability of USP13 on METTL3 was inhibited by Spautin-1. Interestingly, a significant reduction in the half-life of METTL3 protein was found following cotreatment with Spautin-1 and CHX (Fig. 8a). Further, Spautin-1 treatment reduced METTL3 and ATG5 protein levels in 143B cells while this effect was counteracted by MG132, indicating that Spautin-1, which acts in a similar way to silencing of USP13, accelerates METTL3 degradation (Fig. 8b). Moreover, treatment of Spautin-1 almost abolished the ability of USP13 to cleave ubiquitin moieties from polyubiquitinated METTL3 in 143B cells and HEK 293T cells (Fig. 8c, d).

Next, we examined the therapeutic effects of Spautin-1 on tumor progression *in vivo*. We found that mice receiving Spautin-1 exhibited decreased tumor proliferation and metastasis compared with vehicle-treated mice (Fig. 8e-i). Also, EMT was inhibited in mice receiving Spautin-1 (Fig. 8j). Collectively, our results demonstrated that pharmacological inhibition of USP13 effectively inhibit the malignancy of OS by promoting METTL3 destabilization.

## Discussion

In this present study, by using several loss- and gain-of-functional experiments *in vitro* and *in vivo*, we demonstrated a previously-unreported role of USP13 in promoting glycolysis and progression in OS. USP13 was shown to positively promote glycolysis and tumor progression by stabilizing and deubiquitinating METTL3, which is a well-known “writer” for m^6^A modification, at K488 by removing K48-linked ubiquitin chains. Then, by using RNA-Seq, MeRIP-Seq and various *in vitro* experiments, we found that METTL3 stabilized the autophagy-related gene ATG5 by inhibiting its RNA decay on an IGF2BP3 dependent manner. Pharmacologic inhibition of USP13 by Spautin-1, a small-molecule inhibitor of USP13, showed therapeutic effects on OS. Collectively, this study indicated a role of the USP13-METTL3-ATG5 cascade in OS (Fig. 8k).

Accumulating evidence has suggested the important role of DUBs in various tumors. For example, Chen et al. reported that USP9X could deubiquitinate ALDH1A3 in glioblastoma^22^. Tu et al. reported that USP3 deubiquitinates Claspin, thus activating ATR-Chk1 signaling in tumor resistance to radiation^23^. Park et al. suggested the tumor suppression role of USP11 may be exerted by stabilizing PTEN^24^. Interestingly, the findings of another study indicated that USP11 promotes growth and metastasis by stabilizing PPP1CA in colorectal cancer, suggesting that DUBs could have opposite functions via regulating different proteins in diverse diseases^25^. To date, no DUBs have been studied in OS and their roles are still unclear. In present study, we found that the DUB USP13 was markedly upregulated in OS samples and associated with poor outcome. Using various *in vitro* and *in vivo* functional experiments, USP13 was demonstrated to promote glycolysis and tumor progression in OS. USP13 is a DUB and plays key roles in various biological processes including tumor promotion or inhibition, inflammation, apoptosis, drug resistance and anti-viral responses by cleaving ubiquitin molecules from associated substrates^26^-^32^. Zhang et al. reported that USP13 stabilizes PTEN protein and suppresses human breast cancer^26^. Interestingly, another two studies demonstrated that USP13 could promote tumor progression by deubiquitinating Myc, ACLY and OGDH^31^, ^32^, indicating the opposite role of USP13 in tumor progression or inhibition. In addition, USP13 has been reported to regulate the cellular antiviral response by deubiquitinating STING^28^. Li et al. proposed the anti-inflammatory role of USP13 as a positive regulator of Sigirr stability^30^. In present study, we found that USP13 promotes glycolysis and progression in OS.

To further explore the underlying mechanisms and protein-protein interactions by which USP13 promotes proliferation and metastasis of OS cells, IP/MS was performed and METTL3 was identified as a putative protein that binds to USP13. METTL3, serving as the crucial component of the N^6^-methyltransferase complex, has been demonstrated to be involved in many biological processes by regulating its mRNA targets expression via m^6^A modification^33^. In recent years, research into m^6^A has become a hot-spot in the field of tumor biology^34^-^36^. However, the exact functional role of METTL3 in tumor promotion or inhibition is still controversial. Shen et al. reported that METTL3 functions as an oncogenic protein in colorectal cancer progression by stabilizing HK2 and GLUT1^37^. Another study also revealed that METTL3 promotes tumor progression by maintaining SOX2 expression^38^. Furthermore, Lin et al. showed that METTL3 promotes migration, invasion and epithelial-mesenchymal transition in cancer cells^39^. However, several other studies have reported the opposite functional role of METTL3. It was shown that METTL3 suppressed tumor growth and metastasis and enhanced PD-1 blockade therapy by influencing macrophage reprogramming^15^. He et al. revealed that METTL3 suppresses the development of papillary thyroid cancer through m^6^A/c-Rel/IL-8-regulated neutrophil infiltration^40^. These contradictory results suggest the complex functions of METTL3 in tumor microenvironment. In terms of OS, METTL3 might act as an oncogene. For instance, Jiang et al. reported that METTL3 facilitate malignant proliferation of OS cells via stabilizing HDAC5 in an m^6^A dependent manner^41^. Zhou et al. revealed that METTL3 contributes to OS progression through increasing DANCR mRNA stability^42^. Furthermore, silencing METTL3 inhibits proliferation and migration of osteosarcoma by regulating ATAD2^43^. In this study, by using high-throughput RNA-Seq as well as MeRIP-Seq, ATG5 was determined to be a novel m^6^A target of METTL3. The functional role of METTL3 in promoting OS progression was dependent on increasing m^6^A level and stabilizing ATG5 mRNA, thereby activating oncogenic autophagy in OS. Our findings have suggested the potential biological functions of METTL3 in promoting the development of OS, indicating that METTL3 and m^6^A methylation exert broad influences on tumor progression and precision treatment. It is well-recognized that m^6^A modification is a delicate balance, depending on the cooperation of writers, readers and erasers. The m^6^A-regulated mRNAs are recognized by different readers and exhibit diverse biological functions. YTHDF1 promotes gene expression via promoting the translation of target mRNAs; YTHDF2/3 and YTHDC2 are thought to decrease mRNA stability and accelerate mRNA decay, while IGF2BPs enhances mRNA stability^33^. The correlation ship analysis and experiments verification suggested IGF2BP3 might be a key reader protein that collaborates with METTL3 in regulating ATG5 mRNA stability in OS cells. Not only in OS, IGF2BP3 also acts as an oncogene in many other tumors^44^-^46^, and it always plays a positive role in maintaining target mRNA stability.

M^6^A methylation has been widely studied in recent years, but gaps remain in our understanding of how this process is regulated. Several recent studies have shed light on posttranslational modifications of m^6^A writers, readers and erasers. For example, Sun et al. reported that USP5 increases m^6^A methylation by stabilizing ERK-phosphorylated METTL3, thus promoting stem cell differentiation and tumorigenesis^47^. Fang et al. revealed that EGFR/SRC/ERK-stabilized YTHDF2 promoted cholesterol dysregulation as well as invasion of glioblastoma^48^. Another study showed that FBW7 inhibited tumor development by promoting YTHDF2 degradation^49^. In addition, SUMOylation of METTL3 reduces its methyltransferase activity and decreases m^6^A abundance^50^. Another two studies reported that mTORC1 stimulates oncogenesis via m^6^A RNA modification via SAM and WTAP levels as well as the WTAP-MXD2-cMYC axis^51^-^53^. In this study, we found that USP13 interacts with METTL3 and decreases METTL3 ubiquitination, resulting in reduced METTL3 degradation in OS. Collectively, our findings revealed a relationship between posttranslational modifications and m^6^A methylation. Further studies should continue to focus on the posttranslational regulation of m^6^A writers, readers and erasers, which could be an important factor affecting the m^6^A epitranscriptome.

To investigate the underlying mechanism of the USP13/METTL3 cascade in the progression of OS, we performed RNA-Seq and MeRIP-Seq analysis to identify potential mRNA targets. Our results demonstrated that ATG5 is the critical target mRNA of METTL3 in OS. It is well recognized that ATG5 is essential for autophagosome elongation and autophagy activation. Moreover, previous studies indicated that ATG5 is an essential gene in promoting autophagy and malignant behaviors in several tumors including OS^21^, ^54^-^56^. Here, we demonstrated that ATG5 is involved in USP13/METTL3-mediated malignant autophagy in OS. Interestingly, although many researches have proved the malignant role of autophagy in tumor development, other research has indicated autophagic cell death-regulated inhibition of tumor. Thus, autophagy could exert opposite roles in cancer cells development by promoting the survival or leading to cell death of cancer cells^57^-^59^. Previous studies reported an oncogenic role of ATG5 in the progression of OS^20^, ^21^, so it is reasonable to conclude that the USP13/METTL3 cascade promotes OS progression by targeting oncogenic autophagy. Despite this, the diverse roles of autophagy in tumor biology should be explored more comprehensively.

Collectively, our findings in the present study showed that USP13 promotes aerobic glycolysis and OS development by binding to, stabilizing and reducing ubiquitination and degradation of the m^6^A writer METTL3 at K488 by removing K48-linked ubiquitin chains., which subsequently increases the m^6^A modification of ATG5 mRNA and stabilizes it, thus promoting oncogenic autophagy. Further, we evaluated the potential therapeutic effects of pharmacological inhibition of USP13. Collectively, the findings of this study demonstrated the role of the USP13-METTL3-ATG5 cascade in OS and proposed a promising therapeutic target for treating OS.

## Supplementary Material

Supplementary methods and figures.
