# Supplementary material for: Deubiquitinase USP13 regulates glycolytic reprogramming and progression in osteosarcoma by stabilizing METTL3/m6A/ATG5 axis
Source: Int J Biol Sci. 2023 Apr 23;19(7):2289–303. doi: 10.7150/ijbs.82081 (PMC10158027; doi:10.7150/ijbs.82081)
Supplement: Supplementary file 1 — Supplementary methods and figures. [file ijbsv19p2289s1.pdf]

Supplementary Methods

The shRNA and plasmid construction

The shUSP13 (target sequence-1: GCAGATAAAGAAGTTCACTTT; target sequence-2: GCCAGTATCTAAATATGCCAA) and shMETTL3 (target sequence: GCCTTAACATTGCCCACTGAT) were constructed and cloned into pLKO.1 vector by Genebay Biotech (Nanjing, China). The USP13 (either WT or C345A mutant) expressing plasmids, METTL3 expressing plasmid, ATG5 (either WT or m^6A-mutated (adenosine replaced by cytosine)) expressing plasmids, and a series of Ubiquitin expressing plasmids were all generated by cloning their open reading frame with the N-terminal tag sequence into the vectors (Genebay Biotech, China).

Cell counting kit-8 assay (CCK-8), colony-formation assay and 5-ethynyl-2'-deoxyuridine (EdU) assay

These functional experiments for assessing cell proliferation were conducted as previously described[1,2].

Invasion assay, 3D tumor spheroid cell-invasion assay and chemotaxis assay

These functional experiments for assessing cell migration and invasion were conducted as previously described[1,2].

Measurement of extracellular acidification rate (ECAR)

The ECAR was examined using a Seahorse XF96 Metabolic Flux Analyzer (Seahorse Biosciences, USA) according to the manufacturer's protocols. In brief, OS cells (3×10^4) in indicated groups were plated into each well of a Seahorse XF96 cell culture microplate. ECAR was evaluated by sequential adding glucose (10 mM), oligomycin (1 mM) and 2-deoxyglucose (2-DG, 80 mM). Quantification of data was performed using XFe Wave software (Seahorse Biosciences) according to the manufacturer's instructions.

Measurement of glucose consumption, lactate production and ATP production

The culture medium of OS cells was obtained for glucose and lactate assays. Glucose levels were detected using a glucose assay kit (Nanjing jiancheng) and lactate levels were detected using a lactic acid assay kit (Nanjing jiancheng) according to the manufacturer's instructions. The level of intracellular ATP was detected using an ATP assay kit (Beyotime) according to the manufacturer's instructions.

Glucose metabolic flux analysis

^{13}C-Labeled intracellular metabolites were assessed as previously described[3]. In brief, 2×10^7 OS cells were incubated with $^{13}C_6$-labeled glucose (2g/L, Sigma) for 2h. Then, metabolites were collected, and those containing at least one ^{13}C atom were evaluated using an LC system equipped with a TripleTOF 5600 mass spectrometer (SCIEX, Framingham). Concentrations of $^{13}C_6$-labeled metabolites were normalized to cell number.

qRT-PCR

Total RNAs from OS cells and tissues were obtained using TRIzol reagent (TaKaRa Bio Inc., Japan). mRNA was reversely transcripted into cDNA using the PrimeScript RT Master Mix Kit (TaKaRa Bio Inc.) according to the manufacturer's instructions. qRT-PCR was then carried out using the SYBR Green PCR Kit (TaKaRa Bio Inc.) on an ABI7900 fast real-time PCR system (Applied Biosystems, USA) with β-actin as the internal control. The primer sequences used are as follows: USP13 primer: forward 5'-TCTACAAGAACGAGTGCGCC-3' and reverse 5'-TTGGTAACGCTCCACCAGAC-3'; METTL3 primer: forward 5'-ATCCCCAAGGCTTCAACCAG-3' and reverse 5'-GCGAGTGCCAGGAGATAGTC-3'; ATG5 primer: forward 5'-GGGTCCCTCTTGGGGTACAT-3' and reverse 5'-ACCACACATCTCGAAGCACA-3'; β-actin primer: forward 5'-GTCATTCCAAATATGAGATGCGT-3' and reverse 5'-GCTATCACCTCCCCTGTGTG-3'.

Autophagosome evaluation by GFP-mRFP-LC3 and transmission electron microscopy (TEM)

A GFP-mRFP-LC3 lentivirus (Obio, China) was used to evaluate autophagosome. The location as well as quantity of the autophagosomes were assessed using a confocal microscope (LSM710, Zeiss, Germany). Autophagic lysosomes were labeled red while autophagosomes were labeled red and green (yellow fluorescence). TEM was conducted as previously described[2] and images were acquired under an electron microscope (FEI, Hillsboro, OR, USA).

Immunohistochemistry (IHC)

IHC staining of 100 human OS specimens in tissue microarray (TMAs) were conducted using anti-USP13, -METTL3 and -ATG5 antibodies respectively. Samples were fixed in 4% paraformaldehyde followed by embedded in paraffin and was subsequently cut into 4-μm thick sections. Then IHC was performed by incubating sections with the indicated primary antibodies overnight and secondary antibody the next day.

Western blot

We used radioimmunoprecipitation assay (RIPA) lysis buffer (Beyotime) to lyse OS cells. After concentration examination, proteins were separated in SDS-PAGE, transferred onto PVDF membranes (Merck-Millipore), blocked with 5% BSA and incubated with specific primary antibodies at 4 °C overnight. Then, membranes were incubated with corresponding secondary antibodies at RT. Finally, the chemiluminescence reagent (Merck-Millipore) was applied to visualize the reacting bands.

Immunoprecipitation (IP)

A BCA protein assay kit (Beyotime) was used to detect the protein concentrations. Firstly, we used the protein A/G-agarose beads (Santa Cruz Biotechnology, USA) to preclear cell lysates for 1 h. Then the lysates were immunoprecipitated with the indicated antibodies at 4 °C overnight followed by incubation with protein A/G-agarose beads for 2 h at the next day. The immunocomplexes were then washed five times using RIPA lysis buffer and eluted by boiling for western blotting analysis.

***In vivo* ubiquitination assays**

Endogenous METTL3 ubiquitination level was evaluated by immunoprecipitating cell lysates with anti-METTL3 antibody followed by immunoblotted with anti-Ub antibody. Exogeneous METTL3 ubiquitination level was assessed after co-transfecting Flag-tagged USP13 (WT or C345A mutant), Myc-tagged METTL3 and HA-tagged Ub in HEK 293T cells. Lysate proteins were immunoprecipitated and evaluated by immunoblotted with specific antibodies.

Measurement of m^6A modification by LC-MS/MS

Quantification of RNA m^6A modification was conducted by LC-MS/MS as previously described[4]. In brief, 200 ng mRNA and nuclease P1 were incubated in 20 μL buffer containing 25 mM NaCl, 2.5 mM $ZnCl_2$ for 2 h at 37 °C followed by addition of 2.2 μL NH_4HCO_3 (1 M) and alkaline phosphatase, and incubated for 2 h at 37 °C. After centrifugation at 13,000 rpm for 10 min at 4 °C, 10 μL of the solution was further analyzed by LC-MS/MS.

RNA immunoprecipitation (RIP)

METTL3 RIP assay was performed with a Magna RIP™ RNA-Binding Protein Immunoprecipitation Kit (Millipore) according to the manufacturer's instructions. In brief, the cell pellet was lysed in RIP lysis buffer. Then 50 μL protein A/G magnetic beads were incubated with 5 μg anti-METTL3 antibody in RIP wash buffer at RT for 30 min. After that, lysates were

immunoprecipitated with the beads–antibody complex in RIP immunoprecipitation buffer for 4 h at 4 ℃ before 10% RIP lysates were removed for input. IgG was used as a negative control. The magnetic bead-bound complex was centrifuged and washed at least five times, then the immunoprecipitated RNA was analyzed by qPCR.

RNA stability assay

To determine the mRNA stability of OS cells with the indicated treatments, actinomycin D (Sigma) at 5 µg/mL was added. After incubation for the indicated times, total RNA was isolated and the half-life of ATG5 was analyzed.

References

1 Liu, W. *et al.* miR-210-5p promotes epithelial-mesenchymal transition by inhibiting PIK3R5 thereby activating oncogenic autophagy in osteosarcoma cells. *Cell Death Dis* **11**, 93, doi:10.1038/s41419-020-2270-1 (2020).

2 Liu, W. *et al.* TRIM22 inhibits osteosarcoma progression through destabilizing NRF2 and thus activation of ROS/AMPK/mTOR/autophagy signaling. *Redox Biol* **53**, 102344, doi:10.1016/j.redox.2022.102344 (2022).

3 Liu, J. *et al.* Long noncoding RNA AGPG regulates PFKFB3-mediated tumor glycolytic reprogramming. *Nat Commun* **11**, 1507, doi:10.1038/s41467-020-15112-3 (2020).

4 Xu, F. *et al.* FBW7 suppresses ovarian cancer development by targeting the N(6)-methyladenosine binding protein YTHDF2. *Mol Cancer* **20**, 45, doi:10.1186/s12943-021-01340-8 (2021).

Supplementary Figures and Figure legends

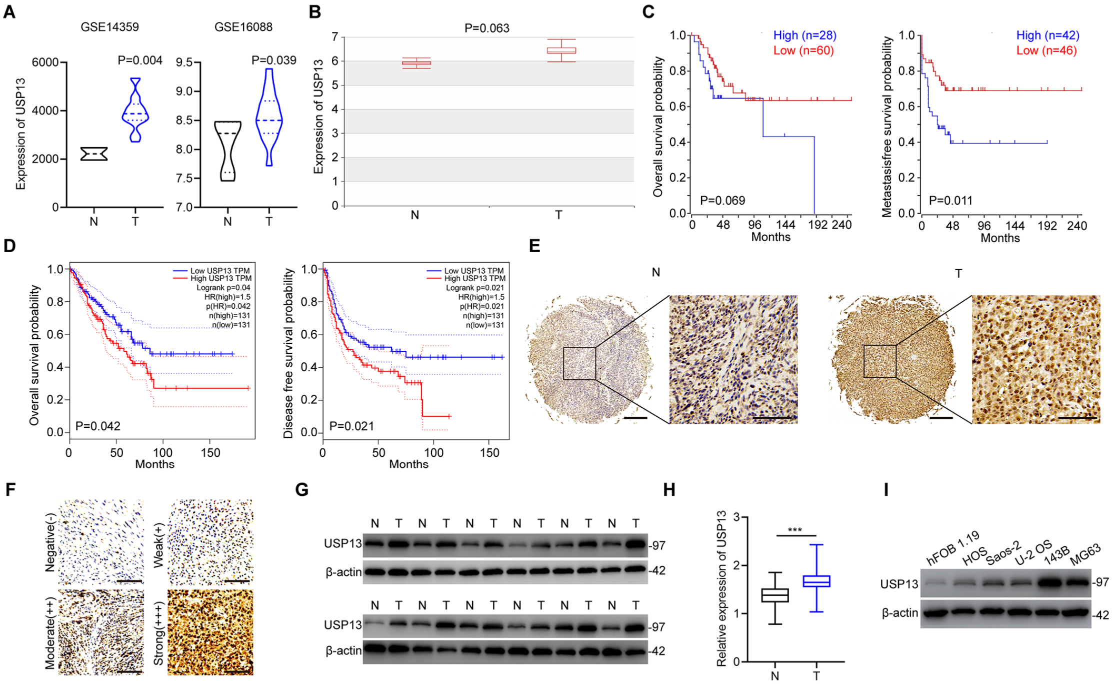

Fig. S1 USP13 is upregulated in OS and associated with a poor outcome. (a) USP13 expression level of OS and adjacent normal tissues from GSE14359 and GSE16088. (b) USP13 expression level from the Human Cancer Metastasis Database (http://hcmdb.i-sanger.com/index, HCMDB). (c) Overall survival and metastasis-free survival of OS patients from an online database (http://hgserver1.amc.nl/cgi-bin/r2/main/cgi). (d) Overall survival and disease-free survival of sarcoma patients in the GEPIA database (http://gepia.cancer-pku.cn/index.html). (e) Representative IHC images of USP13 in OS tissues and matched adjacent normal tissues. Scale bar = 100μm. (f) USP13 expression by IHC staining of OS tissues. Scale bar = 100μm. (g) USP13 expression was evaluated in twelve pairs of OS specimens and adjacent normal specimens via western blot. (h) USP13 mRNA expression was determined in forty pairs of OS specimens and adjacent normal specimens by qRT-PCR. (i) USP13 expression in OS cell lines and the hFOB 1.19 cell line was determined through western blot.

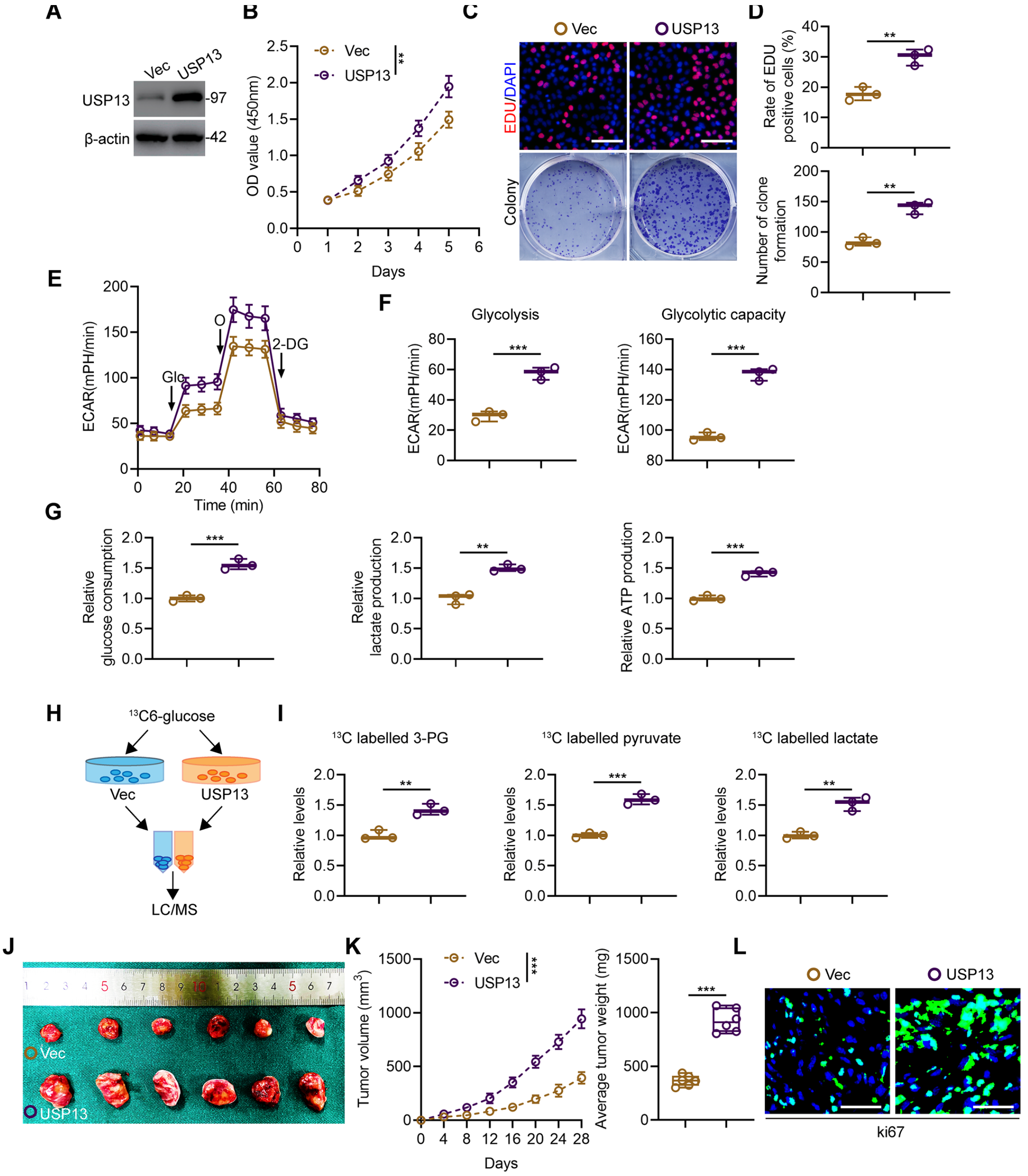

Fig. S2 USP13 promotes glycolysis and cell proliferation in OS. (a) USP13 expression levels after overexpression of USP13 in HOS. (b) Proliferation of HOS cells with USP13 overexpression was determined by CCK-8 assay. (c, d) Representative images and quantifications of mitotic cells and colony number in HOS cells with USP13 overexpression. Scale bar = 200μm. (e) Extracellular acidification rate (ECAR) was measured in USP13-overexpressed HOS cells. (f) Quantification of glycolysis and glycolytic capacity. (g) Glucose consumption, lactate production and ATP production were measured in HOS cells with USP13 overexpression. (h) Flowchart of glucose metabolic flux analysis. (i) ^{13}C-labeled metabolic intermediates of glycolysis were measured in HOS cells with USP13 overexpression. (j) Representative images of xenograft tumors in USP13-overexpreesion

and negative control HOS cells groups. (k) The growth and weight of xenografts tumors in USP13-upregulated and negative control HOS cells groups. (l) Ki67 staining in indicated groups. Scale bar = 50μm.

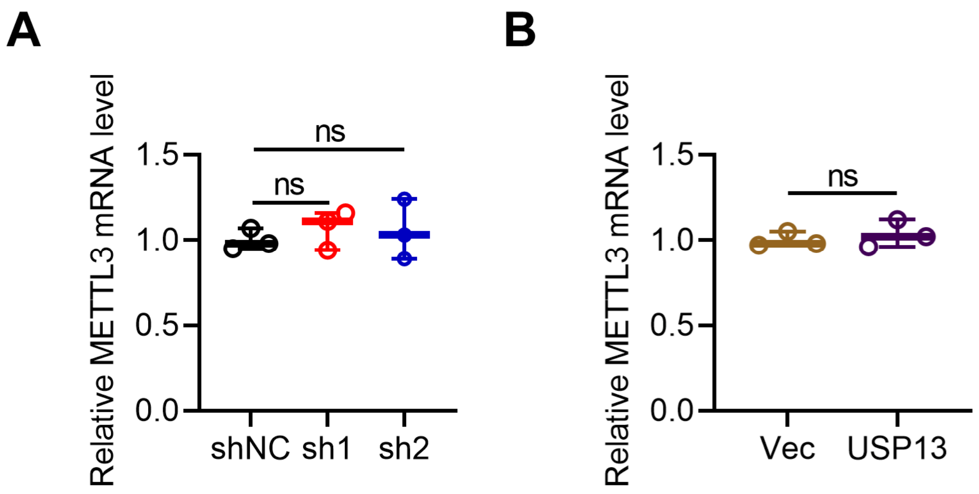

Fig. S3 METTL3 mRNA level in OS cells in the indicated groups. (a) METTL3 mRNA level in 143B cells with or without USP13 depletion. (b) METTL3 mRNA level in HOS cells with USP13 overexpression and corresponding control.

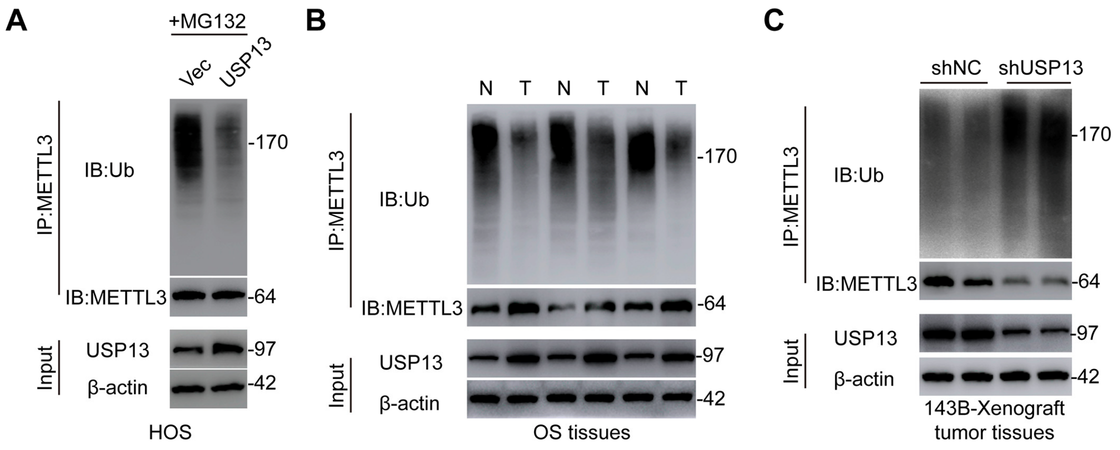

Fig. S4 USP13 inhibits METTL3 ubiquitination. (a) Endogenous METTL3 ubiquitination level in HOS cells with USP13 overexpression or corresponding control. (b) Ubiquitination assay of METTL3 in OS tumor tissues and adjacent normal tissues. (c) Ubiquitination assay of METTL3 in tumors derived from shUSP13 and shNC 143B cells.

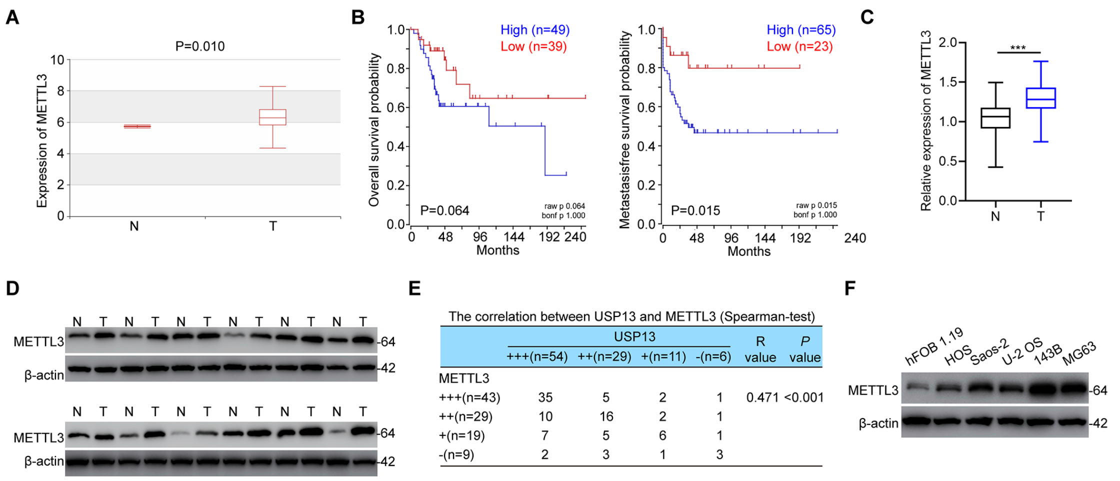

The correlation between USP13 and METTL3 (Spearman-test)

	USP13				R value	*P* value
	+++(n=54)	++(n=29)	+(n=11)	-(n=6)		
METTL3						
+++(n=43)	35	5	2	1	0.471	<0.001
++(n=29)	10	16	2	1		
+(n=19)	7	5	6	1		
-(n=9)	2	3	1	3		

Fig. S5 METTL3 is upregulated and positively correlated with USP13 in OS. (a) METTL3 expression level from the Human Cancer Metastasis Database. (b) Overall survival and metastasis-free survival of OS patients from an online database. (c) METTL3 expression level was detected in forty pairs of OS tissues and matched adjacent normal tissues by qRT-PCR. (d) METTL3 expression level was detected in twelve pairs of OS tissues and matched adjacent normal tissues by western blot. (e) Correlation between USP13 and METTL3 protein expression level in 100 OS tissues using the Spearman test. (f) METTL3 protein level in OS cells and the hFOB 1.19 cell line was assessed.

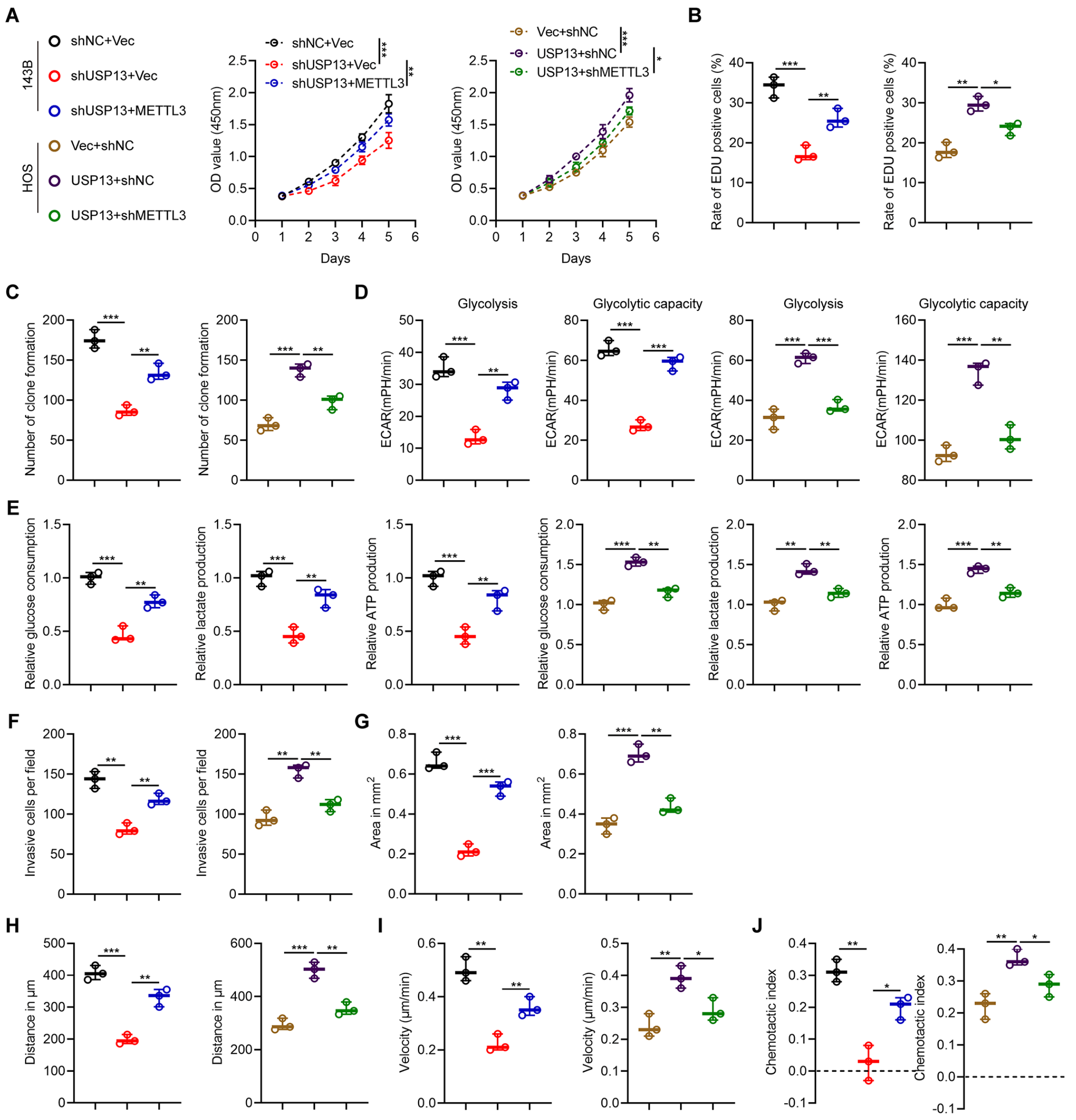

Fig. S6 USP13 promotes glycolysis and progression in OS by interacting with and stabilizing METTL3 *in vitro*. (a–j) Several gain- and loss-of-functional experiments and quantifications including CCK-8 assays (a), EdU assays (b), colony formation assays (c), quantification of glycolysis and glycolytic capacity (d), quantification of glucose consumption, lactate production and ATP production (e), transwell invasion assays (f), 3D tumor spheroid cell-invasion assays (g, h) and chemotaxis assays (i, j) were performed to verify the functional relationship between USP13 and METTL3. Rescue experiments of USP13 knockdown were performed through ectopic expression of METTL3 in 143B cells. Rescue experiments of USP13 overexpression were performed through downregulating METTL3 in HOS cells.

A

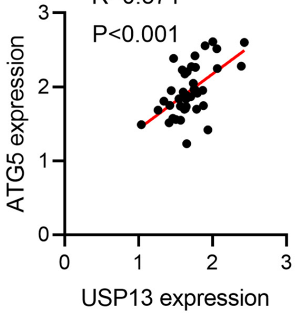

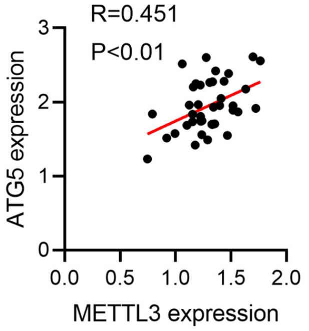

B

The correlation between USP13 and ATG5 (Spearman-test)

	USP13				R value	*P* value
	+++(n=54)	++(n=29)	+(n=11)	-(n=6)		
ATG5						
+++(n=50)	41	6	3	0	0.632	<0.001
++(n=26)	11	14	0	1		
+(n=16)	1	7	6	2		
-(n=8)	1	2	2	3		

The correlation between METTL3 and ATG5 (Spearman-test)

	METTL3				R value	*P* value
	+++(n=43)	++(n=29)	+(n=19)	-(n=9)		
ATG5						
+++(n=50)	35	6	6	3	0.515	<0.001
++(n=26)	6	16	3	1		
+(n=16)	1	5	8	2		
-(n=8)	1	2	2	3		

Fig. S7 Correlation between mRNA and protein expression of USP13 and ATG5, METTL3 and ATG5 in OS tissues.

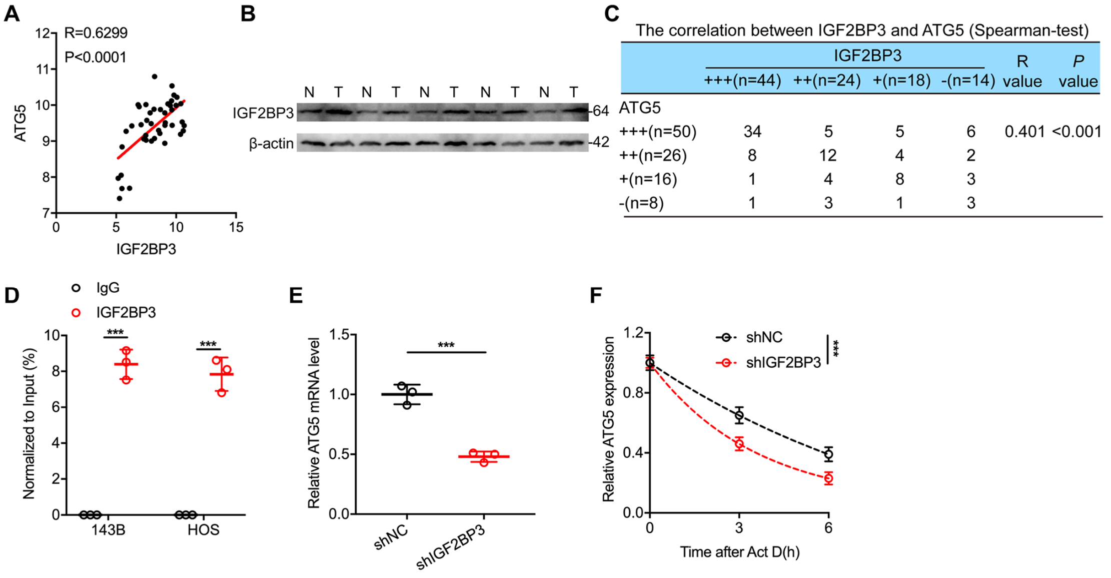

Fig. S8 METTL3 regulates ATG5 mRNA stability on an IGF2BP3-dependent manner. (a) Correlation between mRNA expression of IGF2BP3 and ATG5 in GSE14359 and GSE16088. (b) IGF2BP3 expression level was detected in five pairs of OS tissues and matched adjacent normal tissues by western blot. (c) Correlation between protein expression of IGF2BP3 and ATG5 in OS tissues. (d) IGF2BP3 RIP assays of ATG5 transcripts in IGF2BP3-bound mRNAs in OS cells. (e) ATG5 mRNA expression level in OS cells of indicated groups. (f) RNA lifetime of ATG5 in the indicated OS cells after transcription inhibition (actinomycin D, 5 μg/mL).

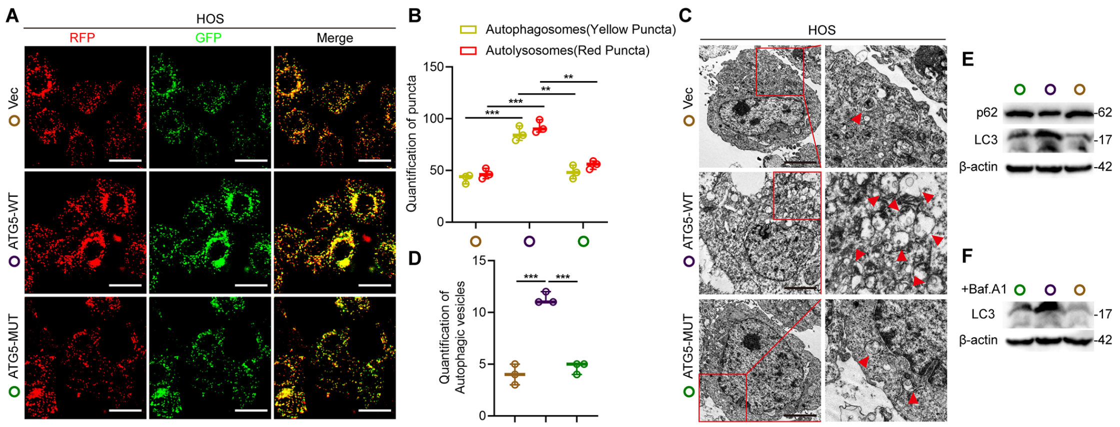

Fig. S9 ATG5-mediated autophagy in OS cells is dependent on its m^{6}A modification. (a, b) HOS cells labelled with GFP-mRFP-LC3 lentivirus were detected, and the cellular puncta were determined in indicated groups. Scale bar = 20μm. (c, d) TEM was used to assess the autophagic microstructure of HOS cells in indicated groups. Scale bar = 2μm. (e) Western blot of LC3 and p62 in transfected HOS cells in the presence or absence of the lysosomal inhibitor Baf.A1.
